# Implementation of non-communicable disease policies: a geopolitical analysis of 151 countries

**DOI:** 10.1016/S2214-109X(19)30446-2

**Published:** 2019-12-05

**Authors:** Luke N Allen, Brian D Nicholson, Beatrice Y T Yeung, Francisco Goiana-da-Silva

**Affiliations:** a Nuffield Department of Primary Care Health Sciences, University of Oxford, Oxford, UK; b Centre of Health Policy, Institute of Global Health Innovation, Imperial College London, London, UK

## Abstract

**Background:**

Most countries have endorsed WHO non-communicable disease (NCD) best buy policies, but we know very little about global implementation patterns and about the geopolitical factors affecting implementation. We aimed to assess global implementation based on analysis of multiple geopolitical datasets.

**Methods:**

We used the 2015 and 2017 WHO NCD progress monitor reports to calculate aggregate implementation scores for 151 countries, based on their implementation of 18 WHO-recommended NCD policies. We ranked all countries and used descriptive statistics to analyse global trends. We used linear regression to assess the associations between policy implementation and World Bank geographic region, risk of premature NCD mortality, percentage of all deaths caused by NCDs, World Bank income group, human capital index, democracy index, and tax burden.

**Findings:**

In 2017, the mean NCD policy implementation score was 49·3% (SD 18·4%). Costa Rica and Iran had the joint-highest implementation scores (86·1% of all WHO-recommended policies). Scores were lowest in Haiti and South Sudan (5·5%). Between 2015 and 2017, aggregate implementation scores rose in 109 countries and regressed in 32 countries. Mean implementation rose for all of the 18 policies except for those targeting alcohol and physical activity. The most commonly implemented policies were clinical guidelines, graphic warnings on tobacco packaging, and NCD risk factor surveys. Our multiple linear regression model explained 61·1% of the variance in 2017 aggregate scores (p<0·0001), but we found evidence of a high degree of collinearity between the explanatory variables.

**Interpretation:**

Implementation of WHO-recommended NCD policies is increasing over time. On average, countries implemented just under half of the NCD policies recommended by WHO in 2017. Nutrition-related policies saw gains, while those related to alcohol and physical activity were the most likely to have been dropped. Aggregate implementation scores tended to be highest in high-income countries that invest in health care and education.

**Funding:**

National Institute for Health Research, Imperial College London, University of Oxford.

## Introduction

Non-communicable diseases (NCDs) are responsible for 73% of all global mortality.^1^ After a slow start, the international community has come to prioritise these conditions, as evidenced by serial High-Level Meetings on NCDs at the UN General Assembly. In 2015, 193 countries committed to reduce premature NCD deaths by one third by 2030, as part of the Sustainable Development Goals. WHO member states have also endorsed a menu of cost-effective NCD best buy policy options that can be used to tackle the pandemic.^2^

In 2015 and 2017, WHO released NCD progress monitor reports^3^, ^4^ that assessed the extent to which 18 NCD policies aligned to the best buys that had been implemented in 151 countries. The reports grouped policies under four time-bound commitments adopted at the second UN High-Level Meeting (appendix pp 2–3). Both reports consist of 151 country profiles and an assessment of whether each of the policies had been implemented fully, partially, or not at all in each country. These assessments were based on national expert opinion, pre-existing data, and policy documents submitted for WHO NCD country capacity surveys. The progress monitors did not provide any global-level analysis of overall policy implementation.

We have detailed global data on the regional and socioeconomic distribution of NCDs and risk factors, as well as clear guidance on which NCD interventions work,^3^, ^5^, ^6^ but there has been much less research on why and where effective policies are implemented around the world. Although both WHO progress monitors presented a wealth of information that could be used to explore implementation patterns, there has been no systematic engagement with this rich dataset except for short regional overviews from Europe and the Caribbean.^7^

Much of our current understanding of why countries do or do not implement particular policies is based on anecdotal evidence rather than quantitative evidence. Common case studies include the USA blocking the inclusion of fiscal measures in the 2018 political declaration on NCDs,^8^ low human and financial capacity hampering NCD policy implementation in sub-Saharan Africa,^9^, ^10^ and high levels of social solidarity facilitating the adoption of personally restrictive policies in Scandinavia.^11^ The underlying factors that are commonly cited as affecting policy implementation include region, NCD burden, human and financial resources, and political ideology and social solidarity.^12^, ^13^, ^14^, ^15^ Understanding what makes countries more likely to introduce effective NCD policies is arguably one of the most important issues in contemporary global health research, and it is vital that rigorous quantitative analyses inform global NCD strategy rather than high-profile, n-of-1 examples.

**Research in context**

**Evidence before this study**

WHO produced its first non-communicable disease (NCD) progress monitor in 2015, providing an assessment of the degree to which 151 countries had implemented 18 NCD policies aligned to the best buy interventions. The report did not synthesise national data to produce regional or global summary data. We searched PubMed for any studies that had previously investigated implementation of NCD policies, using the search terms “implement*” AND (“polic*” OR “intervention”) AND (“chronic disease” OR “noncommunicable” OR “non-communicable”). We applied no date or language filters and we hand-searched references to uncover additional studies.

Our search yielded six studies. A mixed-methods Malawian study from 2016 assessed the extent to which the national NCD plan was being implemented in ten health districts, and found that inadequate human and material resources hampered delivery. A 2018 review of NCD prevention policies in five sub-Saharan countries found slow and uneven implementation with notable gaps around physical activity policies. Document reviews and key informant interviews suggested that factors affecting policy adoption included political commitment, human and financial resources, and industry influence. A 2019 analysis of best buy implementation in seven countries in southeast Asia used WHO NCD progress monitor data to assess regional progress. These countries had weak implementation around diet-related policies, but tended to have fully or partially implemented policies for most of the other domains. The authors identified low levels of institutional capacity, funding, intersectoral coordination, and lack of standardised monitoring and evaluation processes as barriers to full implementation. A 2016 synthesis of African WHO country reports found that most countries had partially implemented most NCD policies in 2011, but rates of implementation were falling over time. Policies around diet and physical activity were most widely implemented, while those pertaining to clinical guidelines and cardiovascular therapies were most commonly overlooked. Human and financial resources, as well as high burdens of other diseases were identified as potential barriers to adoption. Two short regional overviews for the Caribbean and WHO European region were also identified from the grey literature. Both provided basic summaries of NCD progress monitor findings for individual countries, and the European document provided basic summary statistics for the region.

**Added value of this study**

In this geopolitical analysis of 151 countries for which WHO has reported NCD implementation data, we quantified global progress between 2015 and 2017, explored which policies were the most widely implemented, identified countries that were the most and least effective at implementation, and assessed which geopolitical factors were associated with implementation. To our knowledge, this is the first multi-region analysis of NCD policy implementation covering 78% of UN member states, and the first study to quantitively examine national political characteristics and NCD policy implementation rather than health outcomes. On average, countries implemented just under half of the NCD policies recommended by WHO in 2017. Clinical guidelines were the most widely implemented policies and tobacco mass-media campaigns were the least widely implemented. Countries in Europe and central Asia were disproportionately represented among countries with the highest implementation scores, while countries in sub-Saharan Africa were disproportionately represented among countries with the lowest implementation scores. Our multiple linear regression model accounted for around 60% of the variance in policy implementation, using World Bank geographic region, risk of premature NCD mortality, percentage of all deaths caused by NCDs, World Bank income group, human capital index, democracy index, and tax burden as explanatory variables.

**Implications of all the available evidence**

On average, countries implemented just under half of the NCD policies recommended by WHO in 2017, and implementation is slowly improving over time. Market-related policies, especially those related to alcohol and tobacco mass media, were the least widely implemented, along with the provision of cardiovascular therapeutics. Aggregate implementation scores tended to be highest in high-income countries investing in health care and education. Future research should focus on high-achieving outliers and the nature of the relationships between explanatory factors and policy decisions.

Using the data available in the 2015 and 2017 NCD progress monitors, we aimed to answer the following questions: what is the range and mean number of NCD policies that have been implemented globally? Which policies are the most commonly implemented? Which countries have implemented the highest and lowest number of policies? How has the pattern of policy implementation changed over time? Are there differences in the kinds of policies that are adopted or dropped when comparing countries whose overall implementation scores have risen and fallen over time?

We also aimed to explore the extent to which variance in national NCD policy implementation is explained by commonly cited geopolitical factors: region, NCD burden, human and financial resources, and political ideology and social solidarity.

## Methods

### Study design

WHO NCD progress monitors provide epidemiological and policy implementation data for 151 countries. We extracted national-level implementation data using a simple spreadsheet. Following the approach used in an internal WHO memo (unpublished), we accorded a value of one point for each fully implemented intervention, half a point for partially implemented interventions, and zero for interventions that had not been implemented or for which there were no data available. We generated national aggregate scores for 2015 and 2017 and transformed these into percentages so that full implementation of every policy was equal to 100%. For explanatory variables, we used the seven variables presented in table 1. Ethical approval was not required for this study.

**Table 1:** Explanatory variables

	**Data type**	**Description**	**Source**	**Notes**
**Region**				
Geographical region	Categorical	Seven world regions: East Asia & Pacific, Europe & Central Asia, Latin America & Caribbean, Middle East & North Africa, North America, South Asia, and sub-Saharan Africa	World Bank	The most widely used regional classifications in global health are those compiled by WHO and World Bank; we opted for the regional classification used by World Bank because it provides extra detail by breaking the Americas into two regions: North America and Latin America & Caribbean
**Non-communicable disease burden**				
Percentage of deaths caused by non-communicable diseases	Continuous	Percentage of all deaths caused by non-communicable diseases	WHO non-communicable disease Progress Monitor	We used the WHO estimates of the proportion of overall deaths caused by non-communicable diseases and the risk of premature non-communicable disease mortality from the 2015 progress monitor to examine whether baseline non-communicable disease burden was associated with the 2017 score and change in score over time
Risk of premature non-communicable disease mortality	Continuous	Risk of premature non-communicable disease mortality	··	We used the WHO estimates of the proportion of overall deaths caused by non-communicable diseases and the risk of premature non-communicable disease mortality from the 2015 progress monitor to examine whether baseline non-communicable disease burden was associated with the 2017 score and change in score over time
**Human and financial resources**				
Human capital index	Continuous	Composite indicator combining child mortality, stunting, adult survival, expected years of schooling, and harmonised educational test scores	World Bank	We obtained the latest available, 2017 World Bank human capital index scores for each country; a widely used composite measure based on child mortality, stunting, adult survival, expected years of schooling, and harmonised educational test scores
World Bank income group	Ordinal	World Bank income group based on per-capita gross national income (low <US$1045; lower-middle <$4125; upper-middle <$12736; high >$12736)	World Bank	We used the 2017 World Bank analytic classification; this assigns each country to one of four ordinal income groups based on per-capita gross national income
**Political ideology and social solidarity**				
Democracy index	Continuous	Weighted average of 60 items covering civil liberties, pluralism, and political culture	Economist Intelligence Unit	We obtained the latest available, 2017 democracy index data from the Economist Intelligence Unit; these annually produced scores are based on a weighted average of 60 items covering civil liberties, pluralism, and political culture; the scores are well-respected and have been previously used in global health research to analyse access to services
Tax burden	Ordinal	Top, second, third, bottom, and missing data; tripartite composite score with equal weighting accorded to top marginal tax rate on individual income, the top marginal tax rate on corporate income, and the total tax burden as a percentage of GDP	Heritage Foundation	We obtained latest available, 2016 tax burden data from the Heritage Foundation to help distinguish between highly democratic countries that lie on opposing ends of an ideological spectrum that ranges from valuing social solidarity to valuing self-determination (libertarianism); we reasoned that countries that tolerate high top marginal tax rates on individuals and corporations might be more likely to tolerate non-communicable disease policies that constrain free trade and personal choice; the Heritage Foundation is a US think tank with a (right-of-centre) political bias, however their tax burden data are widely respected, transparently composed, and available for many countries

### Statistical analysis

We used simple descriptive statistics to explore policy implementation across the 151 countries. We then did three sets of analyses. First, we examined patterns of implementation among countries with the top 20 and bottom 20 aggregate implementation scores in 2017 with reference to the rest of the world. We calculated 95% CIs of these mean implementation scores for each policy, using a t distribution for the top and bottom 20 (because of the small sample size) and a normal distribution for the rest of the world.

Second, we examined change in aggregate scores between 2015 and 2017. We divided all countries into three groups based on whether their aggregate score had risen, fallen, or remained unchanged between 2015 and 2017 and produced waterfall charts for each group. A new indicator was added for the 2017 NCD progress monitor: effective mass-media campaigns that educate the public about the harms of smoking or tobacco use and second-hand smoke. As some countries may have implemented tobacco mass-media policies before 2015 we discounted this point when analysing changes in aggregate score between 2015 and 2017 (ie, we set the maximum score at 18 points for both years) to allow for fair comparison.

Third, we assessed the extent to which 2017 aggregate policy implementation scores were associated with region, NCD burden, risk of premature NCD mortality, income group, human capital index, democratic index, and tax burden. We used simple linear regression of the aggregate score on each explanatory variable using SPSS software (version 25.0.0.1; IBM, Armonk, NY, USA). We treated tax burden, income group, and world region as categorical variables with bottom tax quartile, low income, and Europe and central Asia as the references. We also used multiple linear regression to create a model that accounted for all of the variables, and to give an estimate of the effect of a given explanatory variable while keeping the others constant.

Normality was checked with residual plots and found to be satisfactory for all variables (data not shown). In all analyses α=0·05.

Since one third of the countries were missing tax data, we treated tax burden data as an ordinal variable, splitting countries into five bins: four quartiles, and one bin for missing data. Once we had transformed the tax burden data into ordinal values, we found that the remaining missing datapoints were clustered in seven countries. We were unable to find alternative estimates for these countries, and felt that the most robust way to treat the missing data was to remove the seven countries with incomplete data from the regression analyses.

To assess the effect of excluding countries that lacked complete data, we re-ran the multiple linear regression model with these countries included. We also re-ran our analysis of high achievers and low achievers using the top and bottom 30 countries to see if this changed the patterns observed, given that 20 was an arbitrary threshold.

### Role of the funding source

The funder of the study had no role in study design, data collection, data analysis, data interpretation, or writing of the report. The corresponding author had full access to all the data in the study and had final responsibility for the decision to submit for publication.

## Results

**Figure 1:**
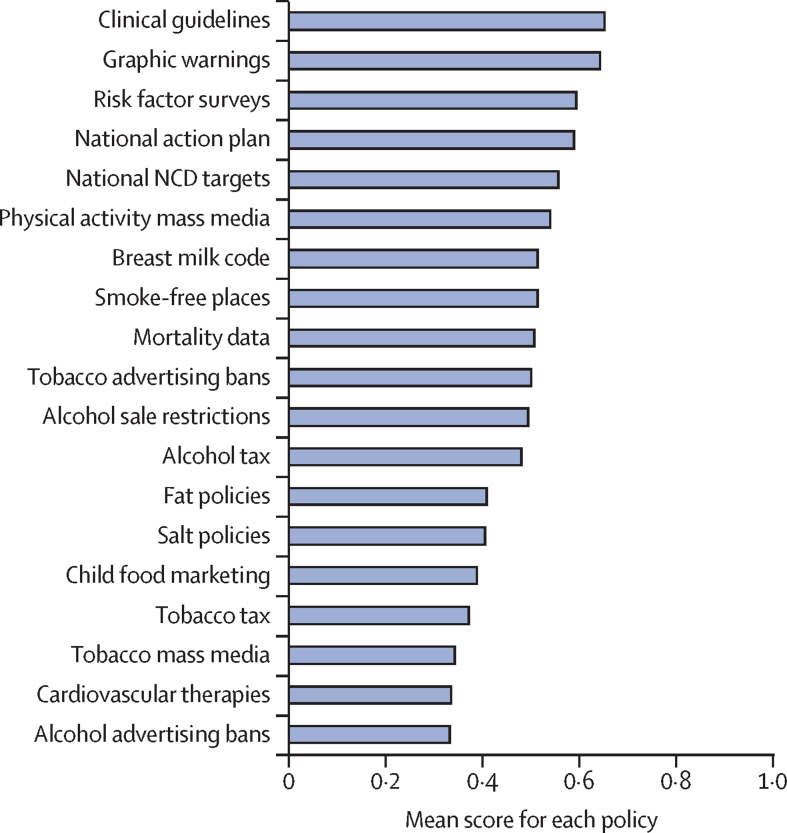
Mean 2017 implementation scores for each NCD policy across all 151 countries A score of 0 means no implementation or no data available. A score of 0·5 means partial implementation. A score of 1 means full implementation. NCD=non-communicable disease.

In this geopolitical analysis of 151 countries, tax burden data were available for 110 of the 151 countries included in the WHO NCD progress monitors. Democracy index scores were available for 144 countries, and risk of premature mortality and proportion of deaths caused by NCDs were available for 147 countries. Policy implementation data were available for all 151 countries, however this was incomplete for 11·4% of policies in 2015 and 3·8% of policies in 2017. Aggregate 2017 NCD policy implementation scores were normally distributed with a slight right skew. Scores ranged from 5·5% to 86·1% (mean 49·3% [SD 18·4%]). The most commonly implemented policies were clinical guidelines, graphic warnings on tobacco packaging, and surveys. The least widely implemented policies were tobacco taxation, tobacco mass-media campaigns, provision of cardiovascular therapies, and alcohol advertising restrictions (figure 1).

Countries in Europe and central Asia were disproportionately represented among the top 20 countries by 2017 aggregate implementation score (table 2). They were weakest around market-related policies such as failure to enforce advertising regulations for breast milk substitutes and—with the exception of graphic warnings on cigarette packaging—measures aimed at constraining the marketing, sale, and consumption of tobacco and alcohol. In comparison with the rest of the world, high-scoring countries were particularly effective at enforcing policies around salt, unhealthy fats, and child marketing, and in providing adequate management of cardiovascular disease (appendix p 4).

**Table 2:** Top and bottom 20 countries by 2017 aggregate implementation score

	**Percentage of all policies implemented**
**Top 20**	
Costa Rica	87%
Iran	87%
UK	82%
Norway	82%
Latvia	79%
Turkey	79%
Bulgaria	79%
Saudi Arabia	79%
Brazil	79%
Estonia	76%
Portugal	76%
Moldova	74%
Lithuania	74%
Slovenia	74%
Thailand	74%
Finland	74%
Russia	74%
Malta	71%
Spain	71%
Italy	71%
**Bottom 20**	
Haiti	5%
South Sudan	5%
Angola	8%
Liberia	11%
Sierra Leone	13%
Burundi	16%
DR Congo	18%
Zimbabwe	18%
Guinea	21%
Mauritania	21%
Nicaragua	21%
Nigeria	21%
Papua New Guinea	21%
Rwanda	21%
Comoros	24%
Côte d'Ivoire	24%
Gabon	24%
Gambia	24%
Zambia	24%
Lesotho	26%

17 of the bottom 20 countries by 2017 aggregate implementation scores were from sub-Saharan Africa. None of the bottom 20 scored any points for interventions around fats, child food marketing, and cardiovascular therapies. The 95% CIs of mean policy implementation scores for the top and bottom 20 countries overlapped in three instances: alcohol sale restrictions, alcohol taxation, and implementing the code for marketing breast milk substitutes (appendix p 4).

In the sensitivity analysis that extended to the top and bottom 30 countries, the patterns of implementation remained largely unchanged. See appendix (pp 14–19) for implementation data for all 151 countries.

**Figure 2:**
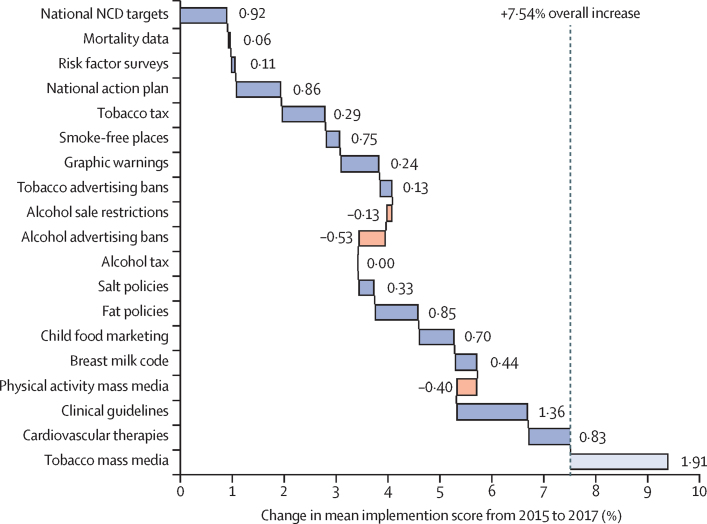
Changes in mean implementation scores for each NCD policy from 2015 to 2017 The waterfall chart shows the relative contribution of each policy to the 7·54% overall increase in mean implementation score from 41·8% in 2015 to 49·3% 2017. Increases are shown in blue, decreases are shown in orange. Tobacco mass-media policy was not present in the 2015 NCD report, and therefore does not contribute to aggregate change in score. NCD=non-communicable disease.

Between 2015 and 2017, mean aggregate implementation scores rose from 41·8% to 49·3%. Gains and losses were fairly evenly distributed across individual policies, however many countries regressed on physical activity mass-media campaigns and restrictions on alcohol sale and promotion (figure 2). The most common gains were related to the introduction of clinical guidelines and therapeutics, setting national targets, and introducing tobacco-related policies.

Aggregate implementation scores rose in 109 countries (72%) of 151 countries, regressed in 32 (21%) countries, and stayed the same in nine (7%) countries. Among the countries with improved scores, gains were evenly spread across policies except for alcohol measures, for which implementation remained relatively flat or declined. Among countries that regressed, rescinded policies clustered around national plans, marketing policies, and physical activity (appendix p 5).

**Figure 3:**
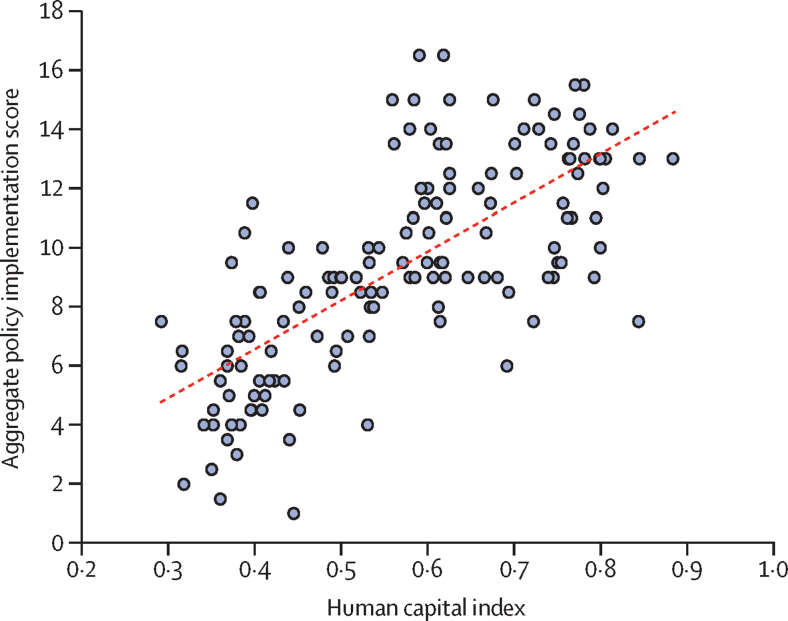
Aggregate 2017 policy implementation scores and human capital index

Overall, there was a positive linear association between 2017 aggregate policy implementation scores and the percentage of deaths caused by NCDs within each country (R^2^=0·53). We found a weak negative association between implementation score and risk of premature NCD mortality (R^2^=0·09). Among the other explanatory variables, human capital indicator had the strongest linear association (R^2^=0·54; figure 3), followed closely by percentage of deaths caused by NCDs (R^2^=0·53). With simple linear regression, all explanatory variables were significantly associated with policy implementation at the α=0·05 level, albeit with varying effect sizes (table 3).

**Table 3:** Regression analyses for 2017 aggregate scores and seven explanatory variables

		**Simple linear regression (unadjusted)**			**Multiple linear regression (adjusted)**	
		Effect (95% CI)	R^2^	p value	Effect (95% CI)	p value
Percentage of deaths caused by non-communicable diseases		0·11 (0·09 to 0·12)	0·53	0·0045	0·02 (−0·04 to 0·08)	0·508
Risk of premature mortality		–0·18 (−0·28 to −0·09)	0·09	0·0098	–0·01 (−0·10 to 0·09)	0·901
Human capital index		16·32 (13·84 to 18·80)	0·54	<0·0001	17·21 (5·84 to 28·59)	0·003
Democracy index		0·71 (0·46 to 0·97)	0·18	0·0039	–0·12 (−0·47 to 0·23)	0·502
Region		··	0·45	0·0010	··	··
	Europe and central Asia	1 (ref)	··	··	1 (ref)	··
	Latin America and Caribbean	–2·13 (−3·58 to −0·68)	0·68	0·004	–0·20 (−2·04 to 1·63)	0·826
	Middle East and north Africa	–0·65 (−2·15 to 0·86)	0·86	0·397	0·78 (−1·11 to 2·67)	0·414
	North America	–0·50 (−3·65 to 2·66)	2·65	0·754	–1·31 (−4·23 to 1·62)	0·379
	South Asia	–1·70 (−4·19 to 0·79)	0·79	0·180	2·44 (−0·40 to 5·28)	0·092
	Sub-Saharan Africa	–5·88 (−7·05 to −4·72)	4·72	0·0091	–0·59 (−3·61 to 2·43)	0·700
	East Asia and Pacific	–1·71 (−3·21 to −0·2)	0·20	0·027	–0·03 (−1·76 to 1·70)	0·973
Tax burden		··	0·37	<0·0001	··	··
	Bottom quartile	1 (ref)	··	··	1 (ref)	··
	Third quartile	0·44 (−1·13 to 2)	··	0·584	0·80 (−0·64 to 2·24)	0·271
	Second quartile	2·73 (1·19 to 4·27)	··	0·001	1·69 (−0·14 to 3·52)	0·070
	Top quartile	1·84 (0·27 to 3·40)	··	0·022	0·27 (−1·74 to 2·28)	0·790
	Missing data	–3·03 (−4·47 to −1·60)	··	0·0084	0·85 (−1·02 to 2·72)	0·369
Income group		··	0·41	<0·0001	··	··
	Low	1 (ref)	··	··	1 (ref)	··
	Lower-middle	1·90 (0·53 to 3·28)	··	0·007	–0·90 (−2·39 to 0·59)	0·235
	Upper-middle	4·43 (3·08 to 5·79)	··	<0·0001	–0·86 (−3·00 to 1·29)	0·430
	High	5·83 (4·54 to 7·11)	··	<0·0001	–1·97 (−4·99 to 1·05)	0·199

With multiple linear regression, only human capital remained significant at the α=0·05 level. Our multiple linear regression model explained 60·3% of the variance in the 2017 aggregate scores (R^2^=0·603, p<0·001; table 3). This means that once the human capital index or percentage of deaths caused by NCDs is known for a given country, adding all of the other variables only explains a further 6–7% of the variance in policy implementation score.

There were weak positive linear associations between policy implementation and democracy index and taxation (appendix pp 6–7), suggesting that more policies were implemented in democratic centre-left countries. These associations were not significant in the adjusted model, possibly because of collinearity with other variables.

The seven countries excluded from the regression analyses due to missing data were Kiribati, Nepal, Seychelles, Solomon Islands, South Sudan, Tonga, and Vanuatu. We note that these are mainly small island developing states. In the sensitivity analysis we re-ran the model on all 151 countries. All variables remained significant with simple linear regression. The overall model explained 61·1% of the variance in 2017 aggregate scores (p<0·001).

The fact that only human capital remained significant at the α=0·05 level suggests that there is a high degree of collinearity between the variables. Collinearity diagnostic testing confirmed that human capital, percentage of deaths caused by NCDs, and Europe and central Asia region all had variance inflation factors of above 10. After re-running the model with these variables removed, R^2^ fell to 55·7% (p<0·0001), all variance inflation factors fell to below 5·5, and three further predictors became significant at the α=0·05 level: sub-Saharan-Africa region (β=–3·72, p<0·01), Latin American and Caribbean region (β=–1·91, p=0·03), and upper-middle income countries (β=0·31, p=0·04; appendix pp 2–3).

Finally, we ran a multiple linear regression model on change in aggregate score between 2015 and 2017. The model explained 18·3% of the variance (p=0·06) and two predictors were significant at the α=0·05 level: Latin America and Caribbean region (p=0·04) and the second tax tier (p=0·049; appendix p 4).

## Discussion

Most countries implemented just under half of WHO-recommended NCD prevention and control policies in 2017. The number of countries that implemented physical activity mass-media campaigns and restrictions on sales and advertising of alcohol fell between 2015 and 2017. There was no change in global implementation of alcohol taxation and mean scores rose for every other NCD policy. Over 70% of countries introduced additional measures between 2015 and 2017. Clinical guidelines, graphic warnings on tobacco packaging, and risk factor surveys were the most commonly implemented policies.

The NCD Countdown 2030 analysis (based on WHO mortality statistics)^16^ revealed wide variability in the rates with which NCD mortality is declining around the world, and suggested that cardiovascular disease is the main driver of premature mortality in low-income and middle-income countries (although we note that weak mortality data collection systems limit confidence in this finding). Our analysis shows that alcohol measures were very poorly implemented, and while graphic warnings on tobacco packaging are widely used, less than a third of countries have fully implemented tobacco taxation and mass-media policies.

Simple linear regression suggested that high-income democratic countries with high levels of human capital and low rates of premature NCD mortality implemented more policies than low-income, undemocratic countries with low levels of human capital and high rates of premature NCD mortality.

Our original multiple linear regression model explained 60·3% of the variance in the 2017 aggregate policy implementation scores. However, we acknowledge that our regression model is a cross-sectional analysis that identifies associations rather than causality. After removing collinear variables the model explained 55·7% of the variance in 2017 aggregate scores. It is not clear which factors account for the remaining 40–50% of variance in scores and, given the lack of empirical research on this topic we can only speculate. The factors included in our model were poor predictors of change in scores between 2015 and 2017.

This study has a number of limitations. Not all policies are equally effective at combating NCDs and our aggregate scores measure breadth of policies rather than effectiveness. According half a point to cover all degrees of partial implementation is imprecise, potentially rendering radically different policy scenarios as equivalent. We were limited by the available data and followed the approach used by WHO in this area.

Between 2015 and 2017 WHO criteria for full and partial implementation changed for several indicators, in all instances making attainment more difficult (appendix pp 5–13). This will have led to underestimation of improvements in the affected policy domains. While it is appropriate that tobacco taxation thresholds increase over time (in line with international legislation), we encourage WHO not to move the goalposts for other policies as it undermines fair appraisal of progress. We also note that the mortality data presented in the progress monitors are several years old in each instance. This limits the utility and reliability of this factor in our regressions.

We accorded a value of zero to all countries where policy data were not available, reasoning that countries with missing data were more likely to have not implemented the policy than to have implemented them in such a way that WHO could not ascertain their existence. This assumption will underestimate mean scores. Fortunately, missing data only accounted for 3·8% of all policy measures in 2017. Missing data were a bigger problem for the tax burden metric. Our decision to transform continuous tax burden into ordinal categories was a statistical trade-off that allowed us to keep every country in the analysis, at the cost of discarding country-level data.

Many of the indicators are self-reported and data quality varies between countries. Although WHO tries to validate the data, in reality little is known about enforcement. Future research should aim to triangulate country data using multiple sources.

Tax burden is an imperfect proxy for centre-right libertarianism; however, we did not find other measures that quantify this domain for such a large number of countries. The Heritage Foundation philosophically oppose high tax rates, although it is not clear if this bias affects their tax burden figures.

Human capital was positively associated with policy implementation and had the greatest explanatory power in simple regression. It is not surprising that countries investing in health care and education are more likely to invest in NCD prevention, nor is it surprising that we found evidence of collinearity for this variable. The World Bank human capital index has been criticised for aggregating weak data from some countries, as well as drawing on a philosophical framing that views social development in terms of economic productivity.^17^, ^18^

Finally, our geopolitical analysis is an exploratory observational study designed to identify geopolitical correlates, not causes. Our contribution is quantitatively testing common assumptions about geopolitical factors that affect policy implementation.

Gravely and colleagues^19^ correlated change in smoking prevalence in 126 countries with implementation of five articles by the WHO Framework Convention on Tobacco Control (FCTC) using a method similar to this study. Hiilamo and Glantz^20^ used regression to assess the association between FCTC ratification and tobacco tax rates for 104 countries, and used the state fragility index^21^ to assess whether implementation was associated with general social and financial development. Juma and colleagues^9^ adopted a qualitative approach to assessing the implementation of NCD prevention policies aligned to WHO best buys, however their work was limited to five African countries. A 2019 analysis by Tuangratananon and colleagues^22^ of best buy implementation in seven countries in southeast Asia used WHO NCD progress monitor data to assess regional progress, alongside other resources. The authors found uneven progress, with implementation gaps largely caused by weak institutional capacity, limited funding, weak intersectoral coordination, and a scarcity of standardised monitoring and evaluation processes. By contrast, our analysis considered macro-level variables on a global rather than regional scale, and carries slightly different policy messages.

Although human and financial resources are helpful, NCD policy implementation is not necessarily expensive. Moldova ranks 130th out of the 151 countries in terms of gross national income, but joint fifth overall for NCD policy implementation. The top aggregate implementation scores were achieved by two middle-income countries: Iran and Costa Rica. NCD policies are highly cost-effective,^3^ and some fiscal policies actually generate revenue.

Nevertheless, high-income countries were over-represented at the top of our implementation table, and crude implementation scores were lowest in low-income countries. Regionally, sub-Saharan countries fared much worse than European countries, even after controlling for social and economic development. Countries that have been lauded for making striking global health gains in recent years, such as Nigeria, Botswana, and Rwanda all came in the bottom ten. This underscores the importance of providing financial and technical support to African countries, especially as they face the highest burdens of premature morbidity and mortality. These findings also highlight avenues for future research, examining the domestic factors that have enabled positive outliers to perform so well. Personal communications suggest that in Moldova, high-level government commitment and intense WHO technical support were important factors in policy implementation.

Previous research has generally found that democracy is positively associated with population health outcomes and service provision.^23^, ^24^ Recent work by Bollyky and colleagues^25^ found that democratisation explained more variance in NCD mortality than GDP in a sample of 170 countries. To our knowledge, our study is the first to examine national political characteristics and NCD policy implementation rather than health outcomes. We found weak evidence to support the common assumption that democracies outperform autocracies, or that centre-left countries outperform more libertarian countries when it comes to implementing NCD policies. In theory, democracies are more responsive to their populations, however they might also be less likely to impose measures that constrain industry profits, or are construed as limiting personal freedoms.

Looking at countries with reputations for social solidarity versus free-market capitalism, Norway had the joint-second highest aggregate implementation score and Finland came joint fifth, but Denmark, Sweden, Iceland, and the Netherlands did not reach the top 20. The USA performed poorly on market-related policies and came 50th out of 151 overall, however other countries with business-friendly reputations performed well—Singapore came 27th, Ireland 26th, and the UK came joint second alongside Norway.

On average, countries implemented just under half of the NCD policies recommended by WHO in 2017, and implementation is slowly improving over time. Market-related policies, especially those related to alcohol and tobacco mass media, were the least widely implemented, along with the provision of cardiovascular therapeutics. Future research is needed to examine regional and domestic factors that affect implementation, focusing on the over-performing and under-performing countries identified by our analysis.
