# Supplementary material for: Implementation of non-communicable disease policies: a geopolitical analysis of 151 countries
Source: Lancet Glob Health. 2019 Dec 5;8(1):e50–8. doi: 10.1016/S2214-109X(19)30446-2 (PMC7024987; doi:10.1016/S2214-109X(19)30446-2)
Supplement: Supplementary appendix [file mmc1.pdf]

THE LANCET
Global Health

Supplementary appendix

This appendix formed part of the original submission and has been peer reviewed. We post it as supplied by the authors.

Supplement to: Allen LN, Nicholson BD, Yeung BYT, Goiana-da-Silva F. Implementation of non-communicable disease policies: a geopolitical analysis of 151 countries. *Lancet Glob Health* 2019; published online Dec 5. http://dx.doi.org/10.1016/S2214-109X(19)30446-2.

Supplementary File

Page 2

Tests for colinearity

Page 4

Multiple linear regression model on change in aggregate score between 2015 and 2017

Page 5

Summary of WHO progress monitor indicator changes between 2015 and 2017

Page 7

Full achievement criteria in 2015 and 2017, taken from the progress monitor appendices

Page 14

Supplementary table: NCD policy implementation in 2015 and 2017

Test for collinearity

Coefficients[a]

Model		Unstandardized Coefficients		Standardized Coefficients	t	Sig.	Collinearity Statistics	
		B	Std. Error	Beta			Tolerance	VIF
1	(Constant)	11.092	1.750		6.339	.000		
	Risk prem NCD mort	-.026	.049	-.043	-.524	.601	.514	1.946
	Democracy Index	.205	.169	.122	1.217	.226	.346	2.889
	Latin America & Caribbean	-1.907	.861	-.184	-2.213	.029	.500	2.001
	Middle East & North Africa	.085	.987	.008	.086	.931	.418	2.390
	North America	-1.768	1.546	-.072	-1.144	.255	.871	1.148
	South Asia	.608	1.410	.032	.431	.667	.637	1.570
	Sub-Saharan Africa	-3.717	1.095	-.467	-3.395	.001	.182	5.483
	East Asia & Pacific	-.925	.846	-.085	-1.094	.276	.570	1.753
	Top	-.974	.974	-.107	-1.000	.319	.302	3.307
	Second	.605	.857	.068	.706	.481	.369	2.709
	Bottom	-.539	.763	-.058	-.707	.481	.509	1.965
	Missing data	-.501	.999	-.063	-.501	.617	.215	4.643
	Income group 2	.031	.731	.004	.042	.966	.424	2.359
	Income group 3	-1.855	.913	-.222	-2.031	.044	.288	3.470
	Income group 4	-2.098	1.088	-.240	-1.928	.056	.223	4.486

a. Dependent Variable: 2017 score

VIF = variance inflation factor

‘Top’, ‘Second’, and ‘Bottom’, and ‘Missing data’ = tax groups

With proportion of deaths, HCI, and region2 (Europe and Central Asia) removed:

Model Summary

Model	R	R Square	Adjusted R Square	Std. Error of the Estimate
1	.747[a]	.557	.506	2.4729

a. Predictors: (Constant), Income group 4, Bottom, North America, East Asia & Pacific, Latin America & Caribbean, Risk prem NCD mort, Second, South Asia, Income group 2, Middle East & North Africa, Top, Democracy Index, Missing data, Income group 3, Sub-Saharan Africa

Coefficients[a]

Model		Unstandardized Coefficients		Standardized Coefficients	t	Sig.	Collinearity Statistics	
		B	Std. Error	Beta			Tolerance	VIF
1	(Constant)	11.092	1.750		6.339	.000		
	Risk prem NCD mort	-.026	.049	-.043	-.524	.601	.514	1.946
	Democracy Index	.205	.169	.122	1.217	.226	.346	2.889
	Latin America & Caribbean	-1.907	.861	-.184	-2.213	.029	.500	2.001
	Middle East & North Africa	.085	.987	.008	.086	.931	.418	2.390
	North America	-1.768	1.546	-.072	-1.144	.255	.871	1.148
	South Asia	.608	1.410	.032	.431	.667	.637	1.570
	Sub-Saharan Africa	-3.717	1.095	-.467	-3.395	.001	.182	5.483
	East Asia & Pacific	-.925	.846	-.085	-1.094	.276	.570	1.753
	Top	-.974	.974	-.107	-1.000	.319	.302	3.307
	Second	.605	.857	.068	.706	.481	.369	2.709
	Bottom	-.539	.763	-.058	-.707	.481	.509	1.965
	Missing data	-.501	.999	-.063	-.501	.617	.215	4.643
	Income group 2	.031	.731	.004	.042	.966	.424	2.359
	Income group 3	-1.855	.913	-.222	-2.031	.044	.288	3.470
	Income group 4	-2.098	1.088	-.240	-1.928	.056	.223	4.486

a. Dependent Variable: 2017 score

Multiple linear regression model on change in aggregate score between 2015 and 2017

Model Summary

Model	R	R Square	Adjusted R Square	Std. Error of the Estimate
1	.427[a]	.183	.072	1.8738

a. Predictors: (Constant), Risk prem NCD mort, East Asia & Pacific, Income group 2, Bottom, North America, Latin America & Caribbean, Second, Middle East & North Africa, South Asia, Income group 4, Top, Democracy Index, Missing data, Income group 3, Proporiton of total deaths, Sub-Saharan Africa, HCI

Coefficients[a]

Model		Unstandardized Coefficients		Standardized Coefficients	t	Sig.	95.0% Confidence Interval for B	
		B	Std. Error	Beta			Lower Bound	Upper Bound
1	(Constant)	1.256	3.370		.373	.710	-5.413	7.924
	Latin America & Caribbean	-1.555	.742	-.272	-2.095	.038	-3.024	-.086
	Middle East & North Africa	.473	.766	.079	.617	.538	-1.044	1.989
	North America	-1.634	1.186	-.120	-1.378	.171	-3.980	.713
	South Asia	-1.203	1.150	-.114	-1.046	.298	-3.479	1.073
	Sub-Saharan Africa	-.007	1.225	-.001	-.005	.996	-2.432	2.419
	East Asia & Pacific	-.849	.703	-.141	-1.209	.229	-2.240	.541
	Top	.720	.746	.143	.965	.336	-.756	2.197
	Second	1.324	.665	.270	1.990	.049	.008	2.641
	Bottom	.759	.583	.148	1.301	.196	-.396	1.914
	Missing data	.791	.774	.181	1.022	.309	-.740	2.323
	Income group 2	.688	.643	.154	1.070	.287	-.584	1.960
	Income group 3	.787	.950	.171	.828	.409	-1.093	2.667
	Income group 4	-.250	1.223	-.052	-.205	.838	-2.671	2.171
	HCI	-1.222	4.609	-.100	-.265	.791	-10.344	7.900
	Democracy Index	-.027	.142	-.029	-.190	.850	-.309	.255
	Proporiton of total deaths	.007	.025	.082	.265	.791	-.042	.055
	Risk prem NCD mort	.000	.038	-.001	-.008	.994	-.075	.075

a. Dependent Variable: change in score

'Top', 'Second', and 'Bottom', and 'Missing data' = tax groups

HCI = human capital index

Summary of WHO progress monitor indicator changes between 2015 and 2017

Indicator	Notes
1: Time-bound national targets	For full achievement in 2017, the supply of supporting documentation was required.
3: Risk factor surveys	For full achievement in 2017, data on risk factors "Raised blood glucose/diabetes", "Raised blood pressure/hypertension", and "Overweight and obesity" must be measured and not self-reported; Supporting documentation was also required.
4: Multisectoral national strategy/action plan	For full achievement in 2017, the supply of supporting documentation was required.
5a: Tobacco excise tax	To achieve full achievement in 2017, tax rate must be raised to at least 75% of the price of most sold brand of cigarettes from at least 70% of the retail price in 2015. To achieve partial achievement in 2017, tax rate must be raised to above 51% of the price of the most sold brand of cigarettes from at least 50% in 2015.
5c: Banning of tobacco advertising, promotion and sponsorship	'Large' and 'medium' sized graphic health warnings were specified as having to cover certain areas in 2017 for full and partial achievement; Plain or standardized tobacco packaging was also acceptable in 2017 for meeting 'fully-met' criteria.
5e: Implementation of effective mass media campaigns on the harms of smoking/tobacco use and second hand smoke	A brand new indicator—the implementation of national anti-tobacco mass media campaigns—was created in 2017 for both full and partial achievement.
6c: Pricing policies on alcoholic beverages	For full achievement in 2017, countries must not have tax incentives or rebates for the production of other alcoholic beverages. For partial achievement in 2017, countries were no longer required to adjust the level of taxation for inflation on at least one of the following alcoholic beverages: beer, wine, or spirits.
7a: Adoption of national policies to reduce population salt/sodium consumption	For full achievement in 2017, implemented policies must be targeted at industry product reformulation across the food supply and/or regulation of salt content of food. Implemented policies must also include public awareness programme and nutrition labelling, and supporting document must be supplied.<br>A partial achievement criterion was created for 2017.
7b: Implementation of national policies that limit saturated fatty acids and virtually eliminate industrially produced trans fatty acids in the food supply	For full achievement in 2017, countries were required to supply supporting documentation.

7c: Implementation of policies that reduce the impact on children of the marketing of foods and non-alcoholic beverages high in saturated fats, trans-fatty acids, free sugars, or salt	For full achievement in 2017, countries were required to supply supporting documentation.
7d: Legislation/regulations fully implementing the International Code of Marketing of Breast-milk Substitutes	For full achievement in 2017, countries' national legal measures must also be categorized as "full provisions in law", entailing the encompassment of all or nearly all provisions of the Code and subsequent World Health Assembly (WHA) resolutions. A partial achievement criterion was created for 2017.
8: National public awareness programme for physical activity and/or diet	For full achievement in 2017, countries need only to have implemented national public awareness programme(s) on physical activity, whereas full achievement in 2015 required implementation of national public awareness programmes on both physical activity and diet. Supporting documentation must be supplied for full achievement in 2017.
9: Evidence-based national guidelines/protocols/standards for the management of major NCDs through a primary care approach	For full achievement in 2017, countries were required to supply supporting documentation.

Full achievement criteria in 2015 and 2017, taken from the progress monitor appendices

(*indicates change)

Indicator	2015 fully achieved criteria	2015 partially achieved criteria	2017 fully achieved criteria	2017 partially achieved criteria
1*	If a country responded "Yes" to the questions "Are there a set of national NCD indicators?" and to the sub question "Are there a set of time-bound national targets for these indicators?". Targets must be time-bound, based on the 9 global targets, and need to address NCD mortality, as well as key risk factors in the country and/or health systems.	If the country responded "Yes" to the questions "Are there a set of national NCD indicators?" and to the sub question "Are there a set of time-bound national targets for these indicators?", but the targets do not cover two of the three areas addressed in the 9 global targets (including mortality) or they are not time-bound.	If a country responds "Yes" to the question "Are there a set of time-bound national targets for NCDs based on the 9 voluntary global targets from the WHO Global Monitoring Framework for NCDs?", **and provides the needed supporting documentation**. Targets must be time-bound, based on the 9 global targets, and need to address NCD mortality, as well as key risk factors in the country and/or health systems.	If the country responds "Yes" to the question "Are there a set of time-bound national targets for NCDs based on the 9 voluntary global targets from the WHO Global Monitoring Framework for NCDs ?", but the targets do not cover two of the three areas addressed in the 9 global targets (including mortality) or they are not time-bound.
2	If the country has:<br>• Data from the five most recent reporting years are, on average, at least 70% usable. Usability is calculated as (Completeness (%))*(1-Proportion Garbage)<br>• At least five years of cause-of-death data have been reported to the WHO.<br>• The most recent year of data reported to the WHO is no more than five years old.	If the country does not meet all of the above criteria but has submitted some vital registration data to WHO.	If the country has:<br>• Data from the five most recent reporting years are, on average, at least 70% usable. Usability is calculated as (Completeness (%))*(1-Proportion Garbage)<br>• At least five years of cause-of-death data have been reported to the WHO.<br>• The most recent year of data reported to the WHO is no more than five years old.	If the country does not meet all of the above criteria but has submitted some vital registration data to WHO.
3*	If the country responded "Yes" to each of the following for adults: "Have surveys of risk factors (may be a single RF or multiple) been conducted in your country for all of the following:" "Harmful alcohol use" (**optional**	If the country responded that at least 3, but not all, of the above risk factors are covered, or the surveys were conducted more than 5 years ago but less than 10 years ago.	If the country responds "Yes" to each of the following for adults: "Have surveys of risk factors (may be a single RF or multiple) been conducted in your country for all of the following:" "Harmful alcohol use" (**optional for the Member States where**	If the country responds that at least 3, but not all, of the above risk factors are covered, or the surveys were conducted more than 5 years ago but less than 10 years ago.

	**for Member States according to national circumstances**), “Physical inactivity”, “Tobacco use”, “Raised blood glucose/diabetes”, “Raised blood pressure/ hypertension”, “Overweight and obesity”, and “Salt / Sodium intake”. Additionally, for each risk factor, the country must indicate that the last survey was conducted in the past 5 years (i.e. 2010 or later for the 2015 CCS survey responses) and must respond “Every 1 to 2 years” or “Every 3 to 5 years” to the sub-question “How often is the survey conducted?”.		**there is a ban on alcohol**), “Physical inactivity”, “Tobacco use”, “Raised blood glucose/diabetes”, “Raised blood pressure/hypertension”, “Overweight and obesity”, and “Salt / Sodium intake”. **For risk factors “Raised blood glucose/diabetes”, “Raised blood pressure/hypertension”, and “Overweight and obesity”, the data must be measured, not self-reported.** Additionally, for each risk factor, the country must indicate that the last survey was conducted in the past 5 years (i.e. 2012 or later for the 2017 CCS survey responses) and must respond “Every 1 to 2 years” or “Every 3 to 5 years” to the sub-question “How often is the survey conducted?”. **The country must also provide the needed supporting documentation.**	
4*	If the country responded “Yes” to the questions “Does your country have a national NCD policy, strategy or action plan which integrates several NCDs and their risk factors?” and to the sub-question “ Is it multisectoral?”. Countries also had to respond “operational” to the sub-question “Indicate its stage” and “Yes” to all of the sub-questions pertaining to the 4 main risk factors and 4 main NCDs: “Does it address one or more of the following major	If the country responded “Yes” to the questions “Does your country have a national NCD policy, strategy or action plan which integrates several NCDs and their risk factors?” and to the sub-question “ Is it multisectoral?”. Countries also had to respond “operational” to the sub-question “Indicate its stage” and “Yes” to at least two of the 4 main risk factors and at least two of the 4 main NCDs.	If the country responds “Yes” to the questions “Does your country have a national NCD policy, strategy or action plan which integrates several NCDs and their risk factors?” and to the sub-question “ Is it multisectoral?”. Countries also have to respond “operational” to the sub-question “Indicate its stage” and “Yes” to all of the sub-questions pertaining to the 4 main risk factors and 4 main NCDs: “Does it address one or more of the following major risk factors?”	If the country responds “Yes” to the questions “Does your country have a national NCD policy, strategy or action plan which integrates several NCDs and their risk factors?” and to the sub-question “ Is it multisectoral?”. Countries also have to respond “operational” to the sub-question “Indicate its stage” and “Yes” to at least two of the 4 main risk factors and at least two of the 4 main NCDs

	risk factors?" "Harmful use of alcohol" **(optional for Member States according to national circumstances)**, "Unhealthy diet", "Physical inactivity", "tobacco" (all 4 must have "Yes") and "Does it combine early detection, treatment and care for:" "Cancer", "Cardiovascular diseases", " Chronic respiratory diseases" and "Diabetes" (all 4 must have "Yes").		"Harmful use of alcohol" **(optional for the Member States where there is a ban on alcohol)**, "Unhealthy diet", "Physical inactivity", "tobacco" (all 4 must have "Yes") and "Does it combine early detection, treatment and care for:" "Cancer", "Cardiovascular diseases", " Chronic respiratory diseases" and "Diabetes" (all 4 must have "Yes"). **Country must also provide the needed supporting documentation.**	
5a*	If the country **has set an excise tax at least 70% of the retail price**.	If the country has set an excise tax **at least 50% but less than 70% of the retail price**.	If the country **has total taxes more than 75% of the price of the most sold brand of cigarettes**.	If the country has total taxes **from 51% up to 75% of the retail price**. of the most sold brand of cigarettes
5b	If all public places in the country are completely smoke-free (or at least 90% of the population covered by complete subnational smoke-free legislation).	If three to seven public places are completely smoke-free, or the law allows designated smoking rooms with strict technical requirements in five or more places.	If all public places in the country are completely smoke-free (or at least 90% of the population covered by complete subnational smoke-free legislation).	If three to seven public places are completely smoke-free, or the law allows designated smoking rooms with strict technical requirements in five or more places.
5c*	If the country has **large health warnings** with all appropriate characteristics as detailed.	If there are medium-size warnings with some or all appropriate characteristics, or large warnings were missing some appropriate characteristics	If the country has **plain/ standardized packaging and/or large graphic health warnings** which are defined as covering on average at least 50% of the front and back of the package with all appropriate characteristics as detailed.	If there are medium-size warnings, which **are defined as covering on average between 30 and 49% of the front and back of package**, with some or all appropriate characteristics, or large warnings that are missing some appropriate characteristics.
5d	If the country has a ban on all forms of direct and indirect advertising.	If the country has a ban on national TV, radio and print media, but not on all other forms of direct and/or indirect advertising.	If the country has a ban on all forms of direct and indirect advertising.	If the country has a ban on national TV, radio and print media, but not on all other forms of direct and/ or indirect advertising.
5e*	N/A	N/A	If the country has a campaign conducted with	If the country has a campaign conducted

			at least seven appropriate characteristics including airing on television and/or radio.	with one to six of the appropriate characteristics.
6a	If a licensing system or monopoly exists on retail sales of beer, wine and spirits; Restrictions exist for on- and off-premise sales of beer, wine, and spirits regarding hours and locations of sales and restrictions exist for off-premise sales of beer, wine, and spirits regarding days of sales; and legal age limits for being sold and served alcoholic beverages are 18 years or above for beer, wine, and spirits.	If there are any, but not all, positive responses to the three indicators above.	If a licensing system or monopoly exists on retail sales of beer, wine and spirits; Restrictions exist for on- and off-premise sales of beer, wine, and spirits regarding hours and locations of sales and restrictions exist for off-premise sales of beer, wine, and spirits regarding days of sales; and legal age limits for being sold and served alcoholic beverages are 18 years or above for beer, wine, and spirits.	If there are any, but not all, positive responses to the three indicators above.
6b	If restrictions exist on alcohol advertising for beer, wine, and spirits through all channels; and detection system exists for infringements on marketing restrictions.	If there are restrictions on at least public service/national TV, national radio and billboards but no detection system exists for infringements.	If restrictions exist on alcohol advertising for beer, wine, and spirits through all channels; and detection system exists for infringements on marketing restrictions.	If there are restrictions on at least public service/national TV, national radio and billboards but no detection system exists for infringements
6c*	If excise tax on all alcoholic beverages (beer, wine, and spirits) is implemented; and adjustment of level of taxation for inflation for beer, wine, and spirits is implemented.	If there is excise tax on all alcoholic beverages (beer, wine, and spirits) **and adjustment of the level of taxation for inflation is implemented on at least one of the alcoholic beverages (beer, wine, spirits)**.	If excise tax on all alcoholic beverages (beer, wine, and spirits) is implemented**; If there are no tax incentives or rebates for production of other alcoholic beverages**; and adjustment of level of taxation for inflation for beer, wine, and spirits is implemented.	If there is excise tax on alcoholic beverages (beer, wine, spirits).
7a*	If the country responds "Yes" to the question " Is your country implementing any policies to reduce population salt consumption?"	N/A	If the country responds "Yes" to the question "Is your country implementing any policies to reduce population salt consumption?" **and to the sub-questions** "Are these targeted at: product	If the country responds "Yes" to the question "Is your country implementing any policies to reduce population salt consumption?", and "Yes" to at least one of

			reformulation by industry across the food supply; regulation of salt content of food; public awareness programme; nutrition labelling ? (must have “Yes” to product reformulation by industry across the food supply and/or regulation of salt content of food, and “Yes” to public awareness programme and nutrition labelling”). Country must also provide the needed supporting documentation.	the four sub-questions “Are these targeted at: product reformulation by industry across the food supply; regulation of salt content of food; public awareness programme; nutrition labelling?”.
7b*	If the country responds “Yes” to the question “Is your country implementing any national policies that limit saturated fatty acids and virtually eliminate industrially produced trans-fats (i.e. partially hydrogenated vegetable oils) in the food supply?”.	N/A	If the country responds “Yes” to the question “Is your country implementing any national policies that limit saturated fatty acids and virtually eliminate industrially produced trans-fats (i.e. partially hydrogenated vegetable oils) in the food supply?”, **and provides the needed supporting documentation.**	N/A
7c*	If the country responds “Yes” to the question “Is your country implementing any policies to reduce the impact on children of marketing of foods and non-alcoholic beverages high in saturated fats, trans-fatty acids, free sugars, or salt?	N/A	If the country responds “Yes” to the question “Is your country implementing any policies to reduce the impact on children of marketing of foods and non-alcoholic beverages high in saturated fats, trans-fatty acids, free sugars, or salt?”, **and provides the needed supporting documentation**.	N/A
7d*	If the country **responds “Yes” to the question “Is your country implementing the International Code of Marketing of Breast-**	N/A	If the country is **assessed as having national legal measures categorized as “full provisions in law”,** whereby countries have enacted legislation or	If the country is assessed as having national legal measures categorized as “many provisions in law” or “few provisions in law”,

	**Milk Substitutes through adoption of national laws?".**		adopted regulations, decrees or other legally binding measures encompassing all or nearly all provisions of the Code and subsequent WHA resolutions.	whereby countries have enacted legislation or adopted regulations, decrees or other legally binding measures encompassing many or few provisions of the Code and subsequent WHA resolutions.
8*	If the country responds "Yes**" to at least one of the following questions**: "Has your country implemented any national public awareness programme **on diet** within the past 5 years?" "Has your country implemented any national public awareness programme **on physical activity** within the past 5 years?"	N/A	If the country responds "Yes" **to the following question**: "Has your country implemented any national public awareness programme **on physical activity** within the past 5 years?", **and provides the needed supporting documentation.**	N/A
9*	If national guidelines/protocols/standards exist for all four NCDs (cardiovascular disease, diabetes, cancer and chronic respiratory diseases) which are being partially or fully implemented.	If the country has guidelines/protocols/standards for at least two of the four NCDs (cardiovascular disease, diabetes, cancer and chronic respiratory diseases), but not for all four, and that these are being partially or fully implemented.	If national guidelines/protocols/ standards exist for all four NCDs (cardiovascular diseases, diabetes, cancer and chronic respiratory diseases), **and the country provides the needed supporting documentation**.	If the country has guidelines / protocols / standards for at least two of the four NCDs (cardiovascular diseases, diabetes, cancer and chronic respiratory diseases), but not for all four.
10	If the country reports that more than 50% of primary health care facilities are offering cardiovascular risk stratification for the management of patients at high risk for heart attack and stroke and that all drugs listed [insulin, aspirin, metformin, thiazide diuretics, ACE inhibitors, CC blockers, statins, and sulphonylurea(s)] were	If the country reports that between 25% to 50% of primary health care facilities are offering cardiovascular risk stratification for the management of patients at high risk for heart attack and stroke and that all of the drugs listed were generally available in the primary care facilities of the public health sector.	If the country reports that more than 50% of primary health care facilities are offering cardiovascular risk stratification for the management of patients at high risk for heart attack and stroke and that all drugs listed [insulin, aspirin, metformin, thiazide diuretics, ACE inhibitors, CC blockers, statins, and sulphonylurea(s)] were generally available in the	If the country reports that between 25% to 50% of primary health care facilities are offering cardiovascular risk stratification for the management of patients at high risk for heart attack and stroke and that all of the drugs listed were generally available in the primary care facilities of the public health sector.

	generally available in the primary care facilities of the public health sector.		primary care facilities of the public health sector.	

Supplementary table: NCD policy implementation in 2015 and 2017

2015	National NCD targets	Mortality data	Risk factor surveys	National action plan	Tobacco tax	Smoke-free places	Graphic warnings	Tobacco advertising bans	Alcohol sale restrictions	Alcohol advertising bans	Alcohol tax	Salt policies	Fat policies	Child food marketing	Breat milk code	Physical activity mass media	Clinical guidelines	Cardiovascular therapies	Total (%)
Brazil	1	1	1	1	0	1	1	1	0.5	1	0.5	1	1	1	1	1	1	0.5	86.1
Costa Rica	1	1	1	1	0.5	1	1	0.5	1	0.5	1	1	1	1	1	1	1	0	86.1
United Kingdom	0	1	1	1	0.5	1	0.5	0.5	0.5	0.5	1	1	1	1	1	1	1	1	80.6
Chile	1	1	1	1	0.5	1	1	0.5	0.5	0	0.5	1	0	1	1	1	1	1	77.8
Canada	0	1	1	1	0.5	1	1	0.5	0.5	0.5	0.5	1	1	1	0	1	1	1	75.0
Colombia	1	1	0.5	1	0	1	0.5	1	0.5	0.5	1	1	1	1	1	1	0.5	0	75.0
Iran	1	0.5	0.5	0	0	1	1	1	1	1	1	1	1	1	1	1	not doc	0	72.2
Latvia	0.5	1	0.5	1	0.5	0.5	0.5	0.5	0.5	0.5	0.5	1	1	1	1	1	0.5	1	72.2
Slovenia	0.5	1	1	0	0.5	0.5	0.5	0.5	0.5	1	0.5	1	1	1	1	1	0.5	1	72.2
Bulgaria	1	0.5	0.5	0.5	0.5	1	0.5	0.5	0.5	1	0	1	0	1	1	1	1	1	69.4
Lithuania	1	1	1	1	0.5	0.5	0.5	0.5	0.5	0.5	0.5	0	1	1	1	1	1	no data	69.4
Malta	1	1	1	1	0.5	1	0.5	0.5	0.5	0.5	0	1	1	0	1	1	0	1	69.4
Russian Federation	1	1	1	1	0	1	0.5	1	0.5	1	0.5	1	0	0	1	0	1	1	69.4
Argentina	1	0.5	1	0.5	0.5	1	1	0.5	0.5	0.5	0	1	1	0	1	1	0.5	0.5	66.7
Czech Republic	1	1	1	1	0.5	0.5	0.5	0.5	0.5	1	0.5	1	0	1	0	1	1	no data	66.7
Ecuador	0	0.5	1	1	0.5	1	1	0.5	0.5	0.5	1	1	1	0	1	1	0.5	0	66.7
France	0	1	0.5	1	0.5	0.5	0.5	0.5	0.5	1	1	1	1	1	1	1	no data	no data	66.7
New Zealand	0	1	1	0	0.5	1	1	0.5	0.5	0.5	1	1	1	1	0	0	1	1	66.7
Spain	0	1	1	0	0.5	1	0.5	1	0.5	0.5	0	1	1	1	0	1	1	1	66.7
Australia	0.5	1	1	1	0	1	1	0.5	0.5	0.5	0	1	1	0	1	1	0.5	no data	63.9
Germany	0.5	1	1	0	0.5	0	0.5	0.5	0.5	0.5	0.5	0	1	1	1	1	1	1	63.9
India	1	0.5	0.5	1	0	0.5	0	0.5	0.5	0.5	0.5	1	1	1	1	1	1	0	63.9
Singapore	1	1	1	0	0.5	0.5	1	0.5	0.5	0	0.5	1	1	1	0	1	1	no data	63.9
Afghanistan	0	0	0.5	1	1	0.5	0.5	1	1	1	1	0.5	1	1	1	0	0	0	61.1
Bahrain	1	0.5	0.5	0.5	0	0	0.5	1	no data	no data	no data	1	1	1	1	1	1	1	61.1
Denmark	0	1	1	0	0.5	0	0.5	0.5	0.5	0.5	0.5	1	1	1	1	1	1	no data	61.1
Hungary	1	1	0.5	1	0.5	0.5	0.5	0.5	0.5	1	0	1	1	not doc	not doc	1	0	1	61.1
Ireland	0	1	0.5	0	0.5	1	0.5	0.5	1	0.5	0.5	1	1	1	0	1	1	no data	61.1
Israel	0	1	1	0	0.5	0	0.5	0	0.5	1	1	1	1	1	1	1	0.5	no data	61.1
Jamaica	1	0.5	0.5	1	0	1	1	0	0.5	0	0.5	1	1	1	0	1	1	0	61.1
Kyrgyz Republic	0.5	1	0.5	1	0	0.5	0.5	0.5	0.5	1	0	1	0	1	1	1	1	0	61.1
Malaysia	1	0.5	1	0	0.5	0	1	0.5	0.5	0.5	0.5	not doc	0	1	1	1	1	1	61.1
Portugal	1	1	0.5	no data	0.5	0.5	0.5	0.5	0.5	1	0	1	0	1	1	1	1	0	61.1
Belgium	0.5	1	1	1	0.5	0.5	0.5	0.5	0.5	0.5	0.5	1	0	0	1	1	0.5	no data	58.3
Finland	0.5	1	1	1	0.5	0	0.5	0.5	..5	1	0.5	1	1	not doc	not doc	not doc	1	1	58.3
Italy	0.5	1	1	1	0.5	0.5	0.5	0.5	0.5	1	0	1	no data	1	no data	1	0.5	no data	58.3
Mongolia	1	0.5	1	1	0	1	1	0.5	0.5	0.5	0.5	0	0	0	1	1	0.5	0	55.6
Norway	0	1	0.5	1	0	0.5	0.5	0.5	0.5	0.5	1	1	not doc	1	not doc	1	1	no data	55.6
Sri Lanka	1	0.5	0.5	1	0.5	0.5	1	0.5	0.5	0.5	1	0	0	0	1	1	0.5	0	55.6
Tajikistan	1	0.5	0.5	1	0	0	0	0.5	0.5	0	1	1	1	1	0	1	1	0	55.6
Thailand	1	0.5	1	0	0.5	1	1	0.5	1	1	0.5	0	0	0	not doc	1	1	0	55.6
Iceland	0	1	1	0	0	0	0.5	0.5	0.5	0.5	1	0	1	1	1	1	0.5	no data	52.8
Kazakhstan	0.5	1	0.5	1	0	0.5	0.5	0.5	0.5	0.5	0.5	1	0	0	0	1	1	0.5	52.8
Korea, Rep.	1	1	1	0.5	0.5	0	0.5	0	0.5	0	0.5	1	not doc	1	0	1	1	0	52.8
Madagascar	1	0	0.5	1	0.5	1	1	1	1	0.5	1	not doc	0	0	0	not doc	1	0	52.8
Netherlands	0	1	0.5	1	0.5	0	0.5	0.5	0.5	0.5	0.5	1	1	not doc	not doc	1	not doc	1	52.8
Philippines	0	1	1	1	0.5	0.5	1	0.5	0.5	0.5	0.5	0	0	0	1	1	0.5	0	52.8
Poland	0.5	0.5	0.5	1	0.5	0.5	0.5	0.5	0.5	1	0.5	no data	0	1	1	1	no data	no data	52.8
Turkey	1	0.5	1	0	0.5	1	1	1	0.5	0.5	0.5	1	0	0	0	1	0	no data	52.8
United States of America	1	1	1	1	0	0	0.5	0	0.5	0.5	0.5	1	1	0	0	1	0.5	no data	52.8
Uruguay	0	1	1	0	0	1	1	1	0.5	0	0	1	0	1	1	no data	0	1	52.8

Kuwait	0	1	0.5	0	0	0.5	0.5	0.5	no data	no data	no data	1	1	0	1	1	1	1	50.0
Mexico	0.5	1	0.5	0.5	0.5	0.5	1	0	0.5	0.5	0.5	0	0	1	1	0	1	0	50.0
Moldova	0	1	1	1	0	0.5	0.5	0.5	0.5	0.5	0.5	0	1	1	0	0	1	0	50.0
Nepal	1	0	0.5	1	0	1	1	1	0.5	0	1	0	0	0	1	1	0	0	50.0
Panama	0	1	0.5	1	0	1	1	1	0.5	0.5	1	0	0	0	1	0	0.5	0	50.0
Qatar	0.5	0.5	0.5	0.5	0	0	0.5	0.5	no data	no data	no data	1	1	1	0	1	1	1	50.0
Sweden	0	1	1	0.5	0	0	0.5	0.5	0.5	1	0.5	0	0	0	1	1	0.5	1	50.0
United Arab Emirates	0.5	0.5	0.5	0.5	0	0.5	0.5	1	no data	no data	no data	1	no data	1	1	1	0.5	0.5	50.0
China	1	0.5	1	1	0	0	0.5	0.5	0.5	1	0.5	no data	0	0	0	1	1	0	47.2
Jordan	0	0.5	1	0.5	0.5	0.5	0.5	0.5	0.5	0.5	0.5	not doc	0	0	1	1	0.5	0.5	47.2
Macedonia, FYR	0	1	0	0	0.5	0.5	0.5	0.5	0.5	0.5	0.5	0	0	0	1	1	1	1	47.2
Paraguay	1	0.5	1	1	0	0	0	0	0.5	1	0	1	0	0	1	1	0.5	0	47.2
Saudi Arabia	0	0.5	1	0.5	0	1	0.5	0	1	1	1	1	0	0	0	1	not doc	no data	47.2
Switzerland	0.5	1	1	0	0.5	0	0.5	0	0.5	0.5	0	1	1	not doc	1	1	no data	no data	47.2
Viet Nam	1	0	0.5	1	0	0.5	1	0.5	0.5	0.5	0.5	0	0	0	1	1	0.5	0	47.2
Albania	0	0.5	0.5	0	0	1	0.5	1	0.5	0	0.5	0	0	0	1	1	0.5	1	44.4
Estonia	0.5	1	1	0.5	0.5	0	0.5	0.5	0.5	0.5	0.5	0	0	0	0	1	1	0	44.4
Oman	0	0.5	0.5	0	0	0	0.5	0	1	1	0	1	0	0	1	1	0.5	1	44.4
Algeria	1	0	0.5	0.5	0	0.5	0	0.5	0.5	1	1	0	0	0	1	1	0	no data	41.7
Armenia	1	0.5	1	0.5	0	0.5	0.5	0	0.5	0.5	0.5	0	0	0	1	0	0.5	0.5	41.7
Bosnia and Herzegovina	0	0.5	0.5	1	0.5	0	0	0.5	0.5	0.5	0.5	0	not doc	1	1	1	not doc	no data	41.7
Cyprus	0	1	0.5	1	0.5	0.5	0.5	0.5	0.5	0.5	0	no data	no data	no data	no data	1	no data	1	41.7
Greece	0	0.5	0.5	0	0.5	1	0.5	0.5	0.5	0.5	0	0	1	0	1	1	0	no data	41.7
Kenya	1	0	0.5	1	0	0	0.5	1	0.5	0.5	0.5	0	0	0	not doc	1	1	0	41.7
Serbia	1	1	0.5	0	0.5	0.5	0.5	0.5	0.5	0.5	1	0	0	0	no data	no data	1	no data	41.7
Togo	1	0	0.5	1	0	0.5	0.5	1	0.5	0	1	0	0	0	0	1	0.5	0	41.7
Tunisia	0	0.5	0.5	0	0.5	0	0	0.5	1	1	0.5	not doc	1	0	1	1	no data	no data	41.7
Ukraine	0	1	0	0	0.5	0.5	1	0.5	0.5	0.5	0.5	0	0	0	1	0	1	0.5	41.7
Bangladesh	0	0	0.5	0	0.5	0	1	0.5	0.5	1	1	0	0	0	1	1	0	0	38.9
Dominican Republic	1	0.5	0.5	1	0	0	0	0	0.5	0	0.5	0	0	0	1	1	0	1	38.9
Indonesia	0.5	0	0.5	0	0	0.5	0.5	0	0.5	1	0.5	0	no data	0	1	1	1	0	38.9
Japan	1	1	1	1	0.5	0	0.5	0	0.5	0	0.5	not doc	not doc	0	0	1	not doc	no data	38.9
Montenegro	0	0.5	0.5	1	0.5	0.5	0.5	0.5	0.5	1	0.5	1	0	0	0	0	0	0	38.9
Seychelles	no data	1	0.5	0	0.5	1	1	0.5	0.5	0.5	0.5	0	0	0	not doc	1	not doc	0	38.9
Slovak Republic	0	1	1	0.5	0.5	0.5	0.5	0.5	0.5	0.5	0.5	0	0	0	0	1	not doc	0	38.9
Austria	0	1	0.5	0	0.5	0	0.5	0.5	0.5	0	0	1	1	0	0	1	0	no data	36.1
Azerbaijan	0	0.5	0.5	0	0	0.5	0.5	0.5	0.5	0.5	1	0	0	0	1	1	no data	0	36.1
Egypt	0.5	0.5	0.5	0	1	0.5	1	0.5	0.5	1	0.5	0	0	0	0	0	0	0	36.1
El Salvador	0	0.5	1	0	0	0	1	0.5	0.5	0	0.5	0	0	0	1	1	0.5	0	36.1
Guatemala	1	0.5	0.5	1	0	1	0	0	0.5	0	0	0	0	0	1	0	1	0	36.1
Honduras	0	0.5	0.5	0	0	1	0.5	0.5	0.5	0	1	0	0	0	1	1	0	0	36.1
Iraq	1	0.5	0.5	0.5	0	0.5	0.5	0.5	0.5	0.5	0	0	no data	0	no data	1	0.5	0	36.1
Kiribati	0.5	0.5	0	1	1	0.5	0.5	1	0.5	0	0	not doc	0	not doc	not doc	1	no data	no data	36.1
Romania	0	1	0	0	0.5	0	0.5	0.5	0.5	0.5	1	0	0	0	1	0	1	0	36.1
South Africa	1	0.5	0.5	0	0	0	0	0.5	0.5	0.5	1	1	not doc	not doc	not doc	1	not doc	no data	36.1
Tonga	1	0	0.5	0.5	0.5	0	0.5	0.5	1	0	0.5	0	not doc	not doc	0	1	0.5	0	36.1
Benin	1	0	0.5	1	0	0.5	0.5	0.5	0.5	0	1	0	0	0	0	0	0.5	0	33.3
Myanmar	0	0	0.5	0	0.5	0.5	0	0.5	0.5	0.5	0.5	0	0	0	1	1	0.5	0	33.3
Nicaragua	0	0.5	0	0.5	0	0.5	0.5	0	0.5	0.5	0.5	0	0	0	1	1	0.5	0	33.3
Peru	0	0.5	0.5	0	0	1	1	0	0.5	0.5	0	1	1	0	0	0	0	no data	33.3
Cambodia	0.5	0	1	0	0	0.5	0.5	0.5	0	0	0.5	0	0	0	1	1	0	0	30.6
Croatia	0	1	0.5	0	0.5	0.5	0.5	0.5	0.5	0.5	0	1	0	0	0	0	0	no data	30.6
Guyana	0	0.5	0	1	0	0.5	0	0	0.5	0.5	0.5	0	0	0	1	1	0	0	30.6
Mauritius	no data	1	no data	no data	0.5	0.5	1	1	0.5	0.5	0.5	no data	no data	no data	no data	no data	no data	no data	30.6
Niger	0	0	0.5	1	0	0.5	1	1	0.5	0.5	0.5	0	0	0	0	0	0	0	30.6
Papua New Guinea	0.5	0	0.5	0	0	1	0	0.5	1	0.5	0.5	0	0	0	1	0	not doc	0	30.6

Sudan	0	0	0.5	0	0.5	0	0	0.5	1	1	1	0	0	0	0	0	1	0	30.6
Zambia	1	0	0.5	1	0	0.5	0	0	0.5	0.5	0.5	not doc	0	not doc	not doc	0	1	0	30.6
Ghana	not doc	0	0.5	1	0	0	0.5	1	0.5	0	0.5	0	no data	no data	no data	1	0	no data	27.8
Guinea	0	0	0.5	1	no data	0.5	0.5	1	0.5	0.5	0.5	0	0	0	no data	0	0	0	27.8
Lebanon	0	0	0.5	0.5	0	1	0.5	0.5	no data	no data	no data	0	0	0	1	0	1	0	27.8
Trinidad and Tobago	no data	1	no data	no data	0	1	1	0	1	0.5	0.5	no data	no data	no data	no data	no data	no data	no data	27.8
Yemen	0	0	0	0	0	0.5	0.5	1	1	1	1	0	0	0	not doc	0	0	0	27.8
Zimbabwe	0	0	0.5	0	0	0.5	0	0	0.5	0.5	1	0	0	0	1	1	0	0	27.8
Georgia	0	0.5	0.5	0	0	0.5	0.5	0	0.5	0.5	0.5	0	0	0	0	0	1	0	25.0
Morocco	0.5	0.5	0.5	0	0.5	0.5	0	0.5	no data	no data	no data	0	0	0	not doc	1	0.5	0	25.0
Pakistan	0	0	0.5	0	0	1	0.5	0.5	0.5	1	0.5	0	0	0	no data	no data	0	0	25.0
Solomon Islands	1	0	0.5	1	0	0.5	1	0.5	no data	no data	no data	0	0	0	no data	not doc	not doc	0	25.0
Timor-Leste	1	0	0.5	0	0	0	0	0	0.5	0	0.5	no data	no data	1	no data	1	0	no data	25.0
Gambia	no data	0	0.5	0	0	0	0.5	0.5	0.5	0	1	0	no data	0	not doc	1	0	0	22.2
Luxembourg	no data	1	no data	no data	0.5	0.5	0.5	0.5	0.5	0	0.5	no data	no data	no data	no data	no data	no data	no data	22.2
Mauritania	0.5	0	0.5	0	0	0	0	0	1	1	1	0	0	0	0	0	0	0	22.2
Namibia	no data	0	no data	no data	0	1	1	0.5	1	0	0.5	no data	no data	no data	no data	no data	no data	no data	22.2
Rwanda	not doc	0	1	1	0	0	0	0	0.5	0	0.5	0	0	0	0	1	not doc	0	22.2
Senegal	0	0	1	0	0	0.5	0.5	0.5	0.5	1	0	0	0	0	no data	0	0	no data	22.2
Cameroon	1	0	0	0	0	0.5	0.5	0.5	0.5	0	0.5	no data	no data	no data	no data	no data	no data	no data	19.4
Comoros	0.5	0	0.5	0	0	0.5	0.5	0	0.5	0.5	0.5	0	0	0	no data	0	0	0	19.4
Congo, Rep.	0	0	0	0	0	1	0.5	0.5	0.5	0	0.5	0	0	0	0	0	0.5	0	19.4
DR Congo	no data	0	no data	no data	0	0.5	0.5	0.5	0.5	0.5	1	no data	no data	no data	no data	no data	no data	no data	19.4
Lao PDR	0.5	0	1	0	0	0.5	0.5	0.5	0.5	0	0	0	0	0	0	0	0	0	19.4
Nigeria	0.5	0	0	1	0	0.5	0	0	0.5	0.5	0.5	0	0	0	not doc	0	not doc	0	19.4
Uganda	not doc	0	0.5	0	0	0.5	0.5	0	0.5	0	0.5	0	0	0	not doc	1	not doc	0	19.4
Burkina Faso	0	0	0.5	0	0	1	0.5	0.5	0	0	0.5	0	0	0	not doc	0	0	0	16.7
Chad	no data	0	0	0	0	1	0.5	1	0.5	0	0	0	0	no data	no data	no data	no data	no data	16.7
Ethiopia	not doc	0	1	0	0	0	0.5	0.5	0.5	0	0.5	0	0	0	not doc	no data	0	no data	16.7
Lesotho	not doc	0	0.5	1	0	0	0	0	1	0	0.5	0	0	0	0	not doc	0	0	16.7
Vanuatu	0	0	0.5	0	0	0	1	1	no data	no data	no data	0	0	not doc	0	not doc	0.5	0	16.7
Botswana	no data	0	no data	no data	0.5	0	0	0.5	1	0	0.5	no data	no data	no data	no data	no data	no data	no data	13.9
Gabon	no data	0	no data	no data	0	0.5	0.5	0.5	0.5	0	0.5	no data	no data	no data	no data	no data	no data	no data	13.9
Malawi	not doc	0	0.5	0	0	0	0	0	0.5	0	0.5	0	0	0	not doc	1	not doc	0	13.9
Mali	no data	0	0.5	0	0	0	0.5	0.5	0.5	0	0	0	0	0	not doc	no data	0.5	0	13.9
Burundi	1	0	0	0	0	0	0	0	0.5	0	0.5	0	0	0	not doc	0	not doc	0	11.1
Mozambique	no data	0	no data	no data	0	0	0	0.5	0.5	0	0.5	no data	no data	no data	no data	no data	no data	no data	8.3
Sierra Leone	no data	0	0.5	0	0	0	0	0	0.5	0	0.5	0	0	0	0	0	0	0	8.3
United Republic of Tanzania	no data	0	no data	no data	0	0	0	0.5	0.5	0	0.5	no data	no data	no data	no data	no data	no data	no data	8.3
Angola	0	0	0.5	0	0	0	0	0	0.5	0	0	0	0	0	0	0	not doc	0	5.6
Côte d'Ivoire	no data	0	no data	no data	0	0	0	0	0.5	0.5	0	no data	no data	no data	no data	no data	no data	no data	5.6
Liberia	no data	0	no data	no data	0	0	0	0	0.5	0	0.5	no data	no data	no data	no data	no data	no data	no data	5.6
Haiti	0	0	0	0	no data	0	0	0	no data	no data	no data	0	0	0	0	0	0	no data	0.0
South Sudan	no data	0	no data	no data	no data	0	0	0	no data	no data	no data	no data	no data	no data	no data	no data	no data	no data	0.0

2017	National NCD targets	Mortality data	Risk factor surveys	National action plan	Tobacco tax	Smoke-free places	Graphic warnings	Tobacco advertising bans	Tobacco mass media	Alcohol sale restrictions	Alcohol advertising bans	Alcohol tax	Salt policies	Fat policies	Child food marketing	Breat milk code	Physical activity mass media	Clinical guidelines	Cardiovascular therapies	Total (%)
Costa Rica	1	1	0.5	**1**	0.5	1	1	0.5	1	1	1	1	1	1	0	1	**1**	1	1	**86.8**
Iran	1	0.5	1	**1**	0	1	1	1	0.5	1	1	1	1	1	1	0.5	**1**	1	1	**86.8**
Norway	1	1	0.5	**1**	0.5	1	0.5	0.5	1	0.5	1	0.5	1	1	1	0.5	**1**	1	1	**81.6**
United Kingdom	0.5	1	1	**1**	1	1	1	0.5	1	0.5	0	0.5	1	1	1	0.5	**1**	1	1	**81.6**
Brazil	1	1	1	**1**	0.5	1	1	1	0.5	0.5	0	0.5	1	1	1	1	**1**	1	0	**78.9**
Bulgaria	1	1	0.5	**1**	1	1	1	0.5	0	0.5	1	0	1	1	1	0.5	**1**	1	1	**78.9**
Latvia	1	1	0.5	**1**	1	0.5	1	0.5	0.5	0.5	1	0.5	0.5	1	1	0.5	**1**	1	1	**78.9**
Saudi Arabia	1	0.5	1	**1**	0	0.5	0.5	0.5	0.5	1	1	1	1	1	1	0.5	**1**	1	1	**78.9**
Turkey	1	1	0.5	**0**	1	1	1	1	1	0.5	1	1	1	1	1	0.5	**1**	0.5	0	**78.9**
Estonia	1	1	0.5	**0.5**	1	0	1	0.5	1	0.5	0.5	0.5	1	1	1	0.5	**1**	1	1	**76.3**
Portugal	1	1	1	**1**	0.5	0.5	1	0.5	1	0.5	0.5	0.5	1	0	1	0.5	**1**	1	1	**76.3**
Finland	0	1	1	**1**	1	0	1	0.5	no data	0.5	1	0.5	1	1	1	0.5	**1**	1	1	**73.7**
Lithuania	1	1	0.5	**1**	1	0.5	1	0.5	0	0.5	1	0.5	1	1	1	0.5	**0**	1	1	**73.7**
Moldova	1	1	0.5	**1**	0.5	0.5	1	1	1	0.5	0	1	0.5	1	1	0.5	**0**	1	1	**73.7**
Russian Federation	1	0.5	1	**1**	0.5	1	1	1	0.5	0.5	0.5	0.5	0.5	1	0	0.5	**1**	1	1	**73.7**
Slovenia	0.5	1	0.5	**1**	1	0.5	0.5	0.5	0	0.5	1	0.5	1	1	1	0.5	**1**	1	1	**73.7**
Thailand	1	0.5	1	**1**	0.5	1	1	0.5	1	1	1	0.5	1	0	0	0	**1**	1	1	**73.7**
Georgia	1	0.5	1	**1**	0.5	0.5	0.5	0	0.5	0.5	1	0.5	0.5	1	1	1	**1**	1	0.5	**71.1**
Italy	0.5	1	0.5	**1**	1	0.5	1	0.5	1	0.5	0.5	0.5	1	1	1	0.5	**1**	0.5	0	**71.1**
Jordan	0.5	0.5	0.5	**0.5**	1	0.5	0.5	0.5	1	0.5	1	0.5	1	1	1	0.5	**1**	0.5	1	**71.1**
Malaysia	1	0.5	1	0.5	0.5	0	1	0.5	1	0.5	0.5	0.5	1	1	1	0	1	1	1	**71.1**
Malta	1	1	0.5	**1**	1	1	1	0.5	no data	0.5	0	0.5	1	1	1	0.5	**1**	0	1	**71.1**
Spain	0	1	0.5	**1**	1	1	0.5	1	0	0.5	0	0.5	1	1	1	0.5	**1**	1	1	**71.1**
Canada	0	1	0.5	**1**	0.5	1	1	0.5	0	1	0	0.5	1	1	1	0.5	**1**	0.5	1	**68.4**
Czech Republic	0.5	1	0.5	**1**	1	0.5	1	0.5	0	0.5	1	0.5	0.5	0	1	0.5	**1**	1	1	**68.4**
France	no data	1	0.5	**1**	1	0.5	1	0.5	no data	0.5	1	0.5	1	1	1	0.5	**1**	1	no data	**68.4**
Ireland	0	1	0.5	**0**	1	1	1	0.5	1	1	0	0.5	1	0	1	0.5	**1**	1	1	**68.4**
Israel	0	1	0.5	**1**	1	0.5	0.5	0	0	0.5	0.5	1	1	1	1	0.5	**1**	1	1	**68.4**
Korea, Rep.	1	1	1	**0.5**	0.5	0.5	0.5	0	1	0.5	0	0.5	1	1	1	0.5	**1**	0.5	1	**68.4**
Netherlands	0	1	0.5	**1**	0.5	0	1	0.5	1	0.5	0	0.5	1	1	1	0.5	**1**	1	1	**68.4**
Singapore	0.5	1	0.5	**0.5**	0.5	0.5	1	0.5	1	1	0	1	1	1	1	0	**1**	1	no data	**68.4**
Chile	1	1	0.5	**1**	1	1	1	0.5	0	0.5	0	0.5	1	0	1	0.5	**0**	1	1	**65.8**
Denmark	0	1	0.5	**1**	0.5	0	1	0.5	1	0	0	0.5	1	1	1	0.5	**1**	1	1	**65.8**
Hungary	0	1	0.5	**1**	0.5	0.5	1	0.5	0	0.5	0.5	0.5	0.5	1	1	0.5	**1**	1	1	**65.8**
Mongolia	1	0.5	1	**1**	0	1	1	1	0	0.5	0	0.5	1	1	1	0.5	**1**	0.5	0	**65.8**
Australia	0.5	1	0.5	**0.5**	0.5	1	1	0.5	1	0.5	0	0.5	0.5	1	1	0	**1**	1	no data	**63.2**
Colombia	1	1	0.5	**1**	0	1	0.5	1	1	0.5	0	1	no data	1	0	0.5	**no data**	1	1	**63.2**
Mauritius	0.5	1	0.5	**0**	0.5	0.5	1	1	1	0.5	1	0.5	0.5	1	1	0	**1**	0.5	0	**63.2**
Romania	0	1	0.5	**1**	0.5	1	1	0.5	0.5	0.5	1	0.5	0.5	0	1	0.5	**1**	1	0	**63.2**
United Arab Emirates	1	0.5	0.5	**1**	0	0.5	0.5	1	0.5	no data	no data	no data	1	1	1	0.5	**1**	1	1	**63.2**
Argentina	1	1	0.5	**0.5**	1	1	1	0.5	0.5	0	0.5	0.5	1	1	0	0.5	**0**	0.5	0.5	**60.5**
Azerbaijan	1	0.5	1	**1**	0	0.5	0.5	0.5	0	0.5	0.5	0.5	1	1	1	0.5	**1**	0.5	0	**60.5**
Belgium	0	1	0.5	**0**	1	0.5	1	0.5	0.5	0.5	0	1	0.5	0	1	0.5	**1**	1	1	**60.5**
China	1	0.5	1	**1**	0.5	0	0.5	0.5	1	0.5	1	0.5	1	1	0	0.5	**0**	1	0	**60.5**
Iraq	1	0.5	0.5	**1**	0.5	0.5	0.5	0.5	0.5	1	0.5	0.5	0.5	1	0	0.5	**1**	1	0	**60.5**
Germany	0.5	1	0.5	**1**	0.5	0	1	0.5	0.5	0	0	0	0	1	1	0.5	**1**	1	1	**57.9**
New Zealand	0	1	0.5	**0**	0.5	1	1	0.5	1	0.5	0	0.5	0.5	1	1	0	**0**	1	1	**57.9**
Oman	1	0.5	0.5	**0**	0	0	0.5	0.5	0	1	1	0.5	1	1	0	0.5	**1**	1	1	**57.9**
Seychelles	1	1	0.5	**1**	0.5	1	1	0.5	1	0.5	0.5	0.5	0	0	0	0.5	**1**	0.5	0	**57.9**
Sri Lanka	1	0.5	1	**1**	0.5	0.5	1	0.5	0	1	0.5	0.5	0	0	0	1	**0**	1	1	**57.9**
Switzerland	0	1	0.5	**1**	0.5	0	0.5	0	1	0	0	0.5	0.5	1	1	0.5	**1**	1	1	**57.9**

United States of America	1	1	1	**1**	0	0	0.5	0	1	0.5	0	0.5	1	1	1	0	**1**	0.5	no data	**57.9**
Afghanistan	0	0	0.5	**1**	0	1	0.5	1	0	1	1	1	0.5	1	1	1	**no data**	0	0	**55.3**
Bahrain	1	0.5	0.5	**1**	0	0	0.5	1	0.5	no data	no data	no data	0.5	1	1	1	**1**	no data	1	**55.3**
Ecuador	0	0.5	0.5	**1**	0.5	1	1	0.5	0	1	0.5	0.5	0.5	1	1	0.5	**0**	0.5	0	**55.3**
Kuwait	1	0.5	0.5	**1**	0	0.5	0.5	1	0	no data	no data	no data	0.5	1	0	1	**1**	1	1	**55.3**
Bangladesh	0.5	0	0.5	**1**	1	0.5	1	0.5	1	0.5	1	0.5	0.5	0	no data	0.5	**0**	1	0	**52.6**
India	1	0.5	0.5	**0**	0	0.5	1	0.5	0.5	0.5	0	0	0.5	1	0	1	**1**	1	0.5	**52.6**
Jamaica	1	0.5	0.5	**1**	0	1	1	0	no data	0.5	0	0.5	1	1	0	0	**1**	1	0	**52.6**
Panama	0	1	0.5	**1**	0.5	1	1	1	0.5	0.5	0	0.5	0	0	0	1	**1**	0.5	0	**52.6**
Poland	0	1	0.5	**0.5**	1	0.5	1	0.5	0.5	0.5	1	0.5	0	0	1	0.5	**0**	no data	1	**52.6**
Sweden	0	1	0.5	**0**	0.5	0	1	0.5	0	0.5	1	0.5	1	1	0	0.5	**0**	1	1	**52.6**
Armenia	1	1	1	**1**	0	0.5	1	0	no data	0.5	1	0.5	0	0	0	1	**0**	0.5	0.5	**50.0**
Bosnia and Herzegovina	no data	0.5	0.5	**1**	1	0	0	0.5	no data	0.5	1	0.5	no data	0	1	0.5	**1**	0.5	1	**50.0**
Cyprus	0	0.5	0.5	**0**	1	0.5	0.5	0.5	0.5	0.5	0	0.5	0.5	1	1	0.5	**1**	0.5	no data	**50.0**
Madagascar	0	0	0.5	**1**	1	1	1	1	0	0.5	0.5	0.5	0	no data	no data	1	**no data**	1	0.5	**50.0**
Qatar	1	0.5	0.5	**1**	0	0	0.5	1	no data	no data	no data	no data	0.5	1	1	0.5	**1**	1	0	**50.0**
Serbia	0	1	0.5	**1**	1	0.5	0.5	0.5	0	0.5	0.5	1	0	0	0	0.5	**0**	1	1	**50.0**
Tajikistan	1	0.5	0.5	**1**	0	0	0	0.5	0	0.5	0.5	0.5	1	1	1	0.5	**0**	1	0	**50.0**
Uruguay	1	1	0.5	**0.5**	0.5	1	1	1	0	0.5	0	0.5	0.5	0	1	0.5	**0**	0	no data	**50.0**
Albania	0.5	0.5	0.5	**1**	0.5	1	0.5	1	0.5	0.5	0	0.5	0	0	0	1	**0**	0.5	0.5	**47.4**
Austria	0	1	0.5	**0**	1	0	1	0.5	1	0	0	0.5	0	1	0	0.5	**1**	0	1	**47.4**
Dominican Republic	1	0.5	0.5	**1**	0.5	0.5	0	0	0	0.5	0	1	0	0	0	1	**1**	1	0.5	**47.4**
Egypt	1	0.5	0.5	**0.5**	0.5	0.5	1	0.5	0	1	1	0.5	0.5	0	0	0.5	**0**	0.5	0	**47.4**
El Salvador	0	0.5	1	**1**	0.5	1	1	0.5	1	0.5	0	0.5	0	0	0	0.5	**0**	1	0	**47.4**
Ghana	0	0	0.5	**1**	0	0.5	0.5	1	0	0.5	1	1	0	0	0	1	**1**	1	0	**47.4**
Greece	0	1	0.5	**0**	1	1	1	0.5	0	0.5	0	0.5	1	1	0	0.5	**0**	0.5	no data	**47.4**
Iceland	0	1	0.5	**0**	0.5	0	0.5	0.5	0	0.5	0.5	0.5	0	1	1	0.5	**1**	1	no data	**47.4**
Kazakhstan	0	1	0	**1**	0	0.5	1	0.5	0.5	0.5	1	0.5	0	no data	no data	0.5	**0**	1	1	**47.4**
Kenya	1	0	1	**1**	0.5	0	0.5	1	1	0.5	0.5	0.5	0	0	0	1	**0**	0.5	0	**47.4**
Kyrgyz Republic	1	1	0.5	**1**	0	0.5	1	0.5	no data	no data	no data	no data	1	1	0	0.5	**0**	1	0	**47.4**
Mexico	0.5	1	0.5	**0.5**	0.5	0.5	1	0	no data	0.5	0	0.5	no data	0	1	0.5	**1**	1	0	**47.4**
Morocco	1	0.5	0.5	**0.5**	0.5	0.5	0	0.5	1	0.5	0	0	1	1	0	0	**1**	0.5	0	**47.4**
Nepal	1	0	0.5	**1**	0	1	1	1	1	no data	no data	no data	0	0	0	1	**1**	0.5	0	**47.4**
Peru	0	0.5	0.5	**0.5**	0	1	1	0	0.5	0.5	0	0.5	0.5	1	1	1	**0**	0.5	0	**47.4**
Ukraine	0	1	0	**0**	1	0.5	1	0.5	0	0.5	0.5	1	0	0	0	0.5	**1**	1	0.5	**47.4**
Viet Nam	1	0	1	**1**	0	0.5	1	0.5	1	0.5	0	0.5	0	0	0	1	**0**	1	0	**47.4**
Algeria	1	0	0.5	**0.5**	0	0.5	0	0.5	no data	0.5	1	1	1	0	0	0.5	**1**	0.5	no data	**44.7**
Guatemala	1	1	0.5	**1**	0	1	0	0	0	0.5	0	0.5	0	0	0	1	**1**	1	0	**44.7**
Honduras	1	0.5	0.5	**0**	0	1	0.5	0.5	1	0.5	0	1	0	0	0	0.5	**1**	0.5	0	**44.7**
Indonesia	1	0	0.5	**1**	0.5	0.5	0.5	0	1	0.5	0.5	0	0	0	0	0.5	**1**	1	0	**44.7**
Malawi	no data	0	6	**0**	no data	0	0	0	no data	1	0	0	0	0	0	0.5	**0**	1	0	**44.7**
Philippines	0	0.5	1	**0**	0.5	0.5	1	0.5	1	0.5	0	0.5	0	0	0	1	**1**	0.5	0	**44.7**
Slovak Republic	0	1	0.5	**0.5**	1	0.5	1	0.5	0	0.5	0.5	0.5	no data	0	0	0.5	**1**	0.5	0	**44.7**
South Africa	1	0.5	0.5	**0**	0.5	0	0	0.5	0	0.5	0	0.5	1	1	1	1	**0**	0.5	no data	**44.7**
Vanuatu	1	0	0.5	**1**	0.5	0	1	1	0	0.5	0	1	0.5	0	1	0	**0**	0.5	0	**44.7**
Lao PDR	0.5	0	0.5	**1**	0	1	1	0.5	no data	0.5	1	0.5	0	0	0	0.5	**0**	1	0	**42.1**
Lebanon	0	0	0.5	**0.5**	0	1	0.5	0.5	0	0.5	0	0.5	0	0	0	1	**1**	1	1	**42.1**
Macedonia, FYR	0	1	0.5	**0**	0.5	1	0.5	0.5	0	0.5	0.5	0.5	0	0	0	0.5	**1**	1	0	**42.1**
Trinidad and Tobago	1	0.5	0.5	**0.5**	0	1	1	0	0	0.5	0	0.5	0	0	0	0.5	**1**	no data	1	**42.1**
Chad	1	0	0.5	**1**	0	1	1	1	no data	0.5	0	0.5	0	0	0	0	**1**	0	0	**39.5**
Croatia	0	1	0.5	**0**	1	0.5	0.5	0.5	0	0.5	0	0.5	1	0	0	0.5	**0**	no data	1	**39.5**
Japan	1	1	1	**1**	0.5	0	0.5	0	0	0.5	0	0.5	0.5	0	0	0	**1**	no data	no data	**39.5**
Kiribati	0.5	0.5	1	**0**	0	0.5	0.5	1	1	0.5	0	0.5	0	0	0	0	**1**	0.5	no data	**39.5**
Montenegro	1	0.5	1	**1**	0.5	0.5	0.5	0.5	0	0.5	1	0	0.5	0	0	no data	**0**	no data	0	**39.5**
Pakistan	0	0	0.5	**0**	0.5	1	0.5	0.5	1	0.5	1	0.5	0	0	0	1	**0**	0.5	0	**39.5**
Sudan	1	0	1	**0**	0.5	0	0	0.5	0	1	1	1	0	0	0	0.5	**0**	1	0	**39.5**

Timor-Leste	1	0	0.5	1	0	0.5	0.5	0.5	0.5	0.5	0	0.5	0	0	0	0	1	1	0	39.5
Cameroon	1	0	0.5	0	0	0.5	0.5	0.5	1	0.5	0	0.5	0	0	0	1	1	0	0	36.8
Myanmar	1	0	0.5	0	0	0.5	0.5	0.5	0	0.5	1	0	0	0	0	0.5	1	1	0	36.8
Paraguay	1	0.5	0.5	1	0	0	0.5	0.5	0	0.5	0.5	0.5	0.5	0	0	0.5	0	0.5	0	36.8
Tonga	1	0	0.5	1	0.5	0.5	0.5	0.5	1	no data	no data	no data	0	0	0	no data	1	0.5	0	36.8
Tunisia	0	0.5	0.5	0	0.5	0	0.5	0.5	0.5	no data	no data	no data	1	1	0	0.5	1	0.5	0	36.8
Uganda	0	0	1	0	0.5	1	0.5	1	0	0.5	0	0.5	0	0	0	1	0	1	0	36.8
Congo, Rep.	0	0	0.5	0	0	1	0.5	0.5	0	0.5	0	0.5	no data	no data	1	0	1	1	0	34.2
Guyana	1	1	0.5	1	0	0.5	0	0	0	0.5	0	0.5	0	0	0	0	1	0.5	0	34.2
Mali	0	0	0.5	0.5	0	0	0.5	0.5	0	0.5	0	0	1	1	1	0.5	0	0.5	no data	34.2
Yemen	0	0	0	0	0.5	0.5	0.5	1	0	1	1	1	0	0	no data	1	0	0	0	34.2
Burkina Faso	1	0	0.5	1	0	1	1	0.5	0	0.5	0	0	0	0	0	0.5	0	0	0	31.6
Cambodia	0.5	0	1	0	0	1	1	0.5	1	0	0	0.5	0	0	0	0.5	0	0	0	31.6
Ethiopia	1	0	0.5	0	0	0.5	0.5	0.5	0.5	0.5	0.5	0.5	0	0	0	0	0	1	0	31.6
Luxembourg	0	1	0.5	0	0.5	0	0.5	0.5	0.5	0.5	0	0.5	0	0	0	0.5	1	0	no data	31.6
Niger	0	0	0.5	1	0	0.5	1	1	0	0.5	0.5	0.5	0	0	0	0.5	0	0	0	31.6
Solomon Islands	1	0	1	1	0	0.5	1	0.5	no data	no data	no data	no data	0	0	0	0.5	0	0.5	0	31.6
Benin	0	0	0.5	1	0	0.5	0.5	0.5	no data	0	0	0.5	0	0	0	1	0	1	0	28.9
Botswana	0	0	1	0	0	0	0	0.5	0	1	0	0.5	0.5	0	0	1	0	1	0	28.9
Mozambique	1	0	0.5	1	0	0	0	0.5	0	0.5	0	0	0	0	1	1	0	0	0	28.9
Namibia	1	0	0.5	0	0	1	1	0.5	0	1	0	0.5	0	0	0	0	0	0	0	28.9
Senegal	0	0	0.5	0	0	0.5	1	1	0	0.5	0.5	0.5	0	0	0	0.5	0	0.5	0	28.9
Lesotho	1	0	1	1	no data	0	0	0	no data	0.5	0	0	1	0	0	0	0	0.5	0	26.3
Togo	1	0	0.5	0	0	0.5	0.5	1	0	0.5	0	0.5	0	0	0	0	0	0.5	0	26.3
United Republic of Tanzania	1	0	0.5	1	0	0	0	0.5	no data	0.5	0	0.5	0	0	0	1	0	0	0	26.3
Comoros	0	0	0.5	0	0	0.5	0.5	0	0	0.5	1	0.5	0	0	0	0.5	0	0.5	0	23.7
Côte d'Ivoire	1	0	0	1	0	0	0	0	0.5	0	0	0.5	0	0	0	0.5	1	0	0	23.7
Gabon	1	0	0.5	0	0	0.5	0.5	0.5	0	0	0	0.5	0	0	0	1	0	0	0	23.7
Gambia	0.5	0	0.5	0	0.5	0	0.5	0.5	0	0.5	0	0.5	0	0	0	1	0	0	no data	23.7
Zambia	0	0	0.5	0	0	0.5	0	0	0	0.5	0	0.5	0	0	0	0.5	1	1	0	23.7
Guinea	0	0	0.5	0	0	0.5	0.5	1	0	0.5	0	0	0	0	0	0.5	0	0.5	0	21.1
Mauritania	1	0	0	0	0	0	0	0	no data	1	1	1	0	0	0	0	0	0	0	21.1
Nicaragua	0	0.5	0	no data	0	0.5	0.5	0	0	0.5	0	0.5	0	0	0	0.5	no data	1	0	21.1
Nigeria	1	0	0	0	0	0	0.5	1	no data	0.5	0	0.5	0	no data	no data	0.5	0	0	0	21.1
Papua New Guinea	0.5	0	0.5	0.5	0	1	0	0.5	0	no data	no data	no data	0	0	0	0.5	0	0.5	0	21.1
Rwanda	0	0	0.5	0	0.5	0	0.5	0	no data	0.5	0	0.5	0	0	0	0.5	0	1	0	21.1
DR Congo	0	0	0	0	0	0.5	0.5	0.5	0	0.5	0	1	0	0	0	0.5	0	no data	0	18.4
Zimbabwe	0	0	0	0	0	0.5	0	0	0	0.5	0	0.5	no data	0	no data	1	no data	1	0	18.4
Burundi	1	0	0	0	0	0	0	0	no data	0.5	0	0.5	0	0	0	0.5	0	0.5	0	15.8
Sierra Leone	0	0	0.5	0	0	0	0	0	0.5	0.5	0	1	0	0	0	0	0	0	0	13.2
Liberia	1	0	0.5	0	0	0	0	0	no data	0.5	0	0	0	0	0	0	0	no data	0	10.5
Angola	0	0	0.5	0	no data	0.5	0	0	0	0.5	0	0	0	0	0	0	no data	no data	0	7.9
Haiti	0	0	0	0	0	0	0	0	0	no data	no data	no data	0	0	0	0	0	1	0	5.3
South Sudan	0	0	0	0	0	0	0	0	0	0.5	0.5	0	0	0	0	0	0	0	0	5.3
